# PLL‐g‐HPA Hydrogel Microneedles Loaded With Rhoifolin Target Macrophages to Alleviate Fibroblast Senescence for Diabetic Wound Healing

**DOI:** 10.1002/adhm.71700

**Published:** 2026-09-16

**Authors:** Jiewen Liao, Lin Gan, Li Lu, Lizhi Ouyang, Yuan Xiong, Fawwaz Al‐Smadi, Guandong Dai, Mohammad‐Ali Shahbazi, Bobin Mi, Mengfei Liu, Guohui Liu

**Affiliations:** ^1^ Department of Orthopedics, Union Hospital, Tongji Medical College Huazhong University of Science and Technology Wuhan China; ^2^ Hubei Province Key Laboratory of Oral and Maxillofacial Development and Regeneration Wuhan China; ^3^ Department of Rehabilitation, Tongji Hospital, Tongji Medical College Huazhong University of Science and Technology Wuhan China; ^4^ Department of Orthopedics, Tongji Hospital, Tongji Medical College Huazhong University of Science and Technology Wuhan China; ^5^ Department of Orthopaedics Pingshan District People's Hospital of Shenzhen Pingshan General Hospital of Southern Medical University Shenzhen Guangdong China; ^6^ Department of Biomaterials and Biomedical Technology The Personalized Medicine Research Institute (PRECISION) University Medical Center Groningen University of Groningen Groningen the Netherlands; ^7^ Fuzhou Zhixin Future Pharmaceutical Technology Co., Ltd Fuzhou China

**Keywords:** cellular senescence, diabetic wounds, dermal fibroblast, macrophages, microneedles, oxidative stress

## Abstract

In this study, we demonstrate that during diabetic wound healing, interleukin‐11 (IL‐11) promotes the M1 polarization of macrophages, which subsequently triggers fibroblast senescence through the enhanced release of pro‐inflammatory cytokines IL‐1β and IL‐6. Notably, Rhoifolin (Rho) effectively inhibits IL‐11‐induced M1 polarization, alleviates the secretion of IL‐1β and IL‐6, and concurrently promotes the release of choline, thereby mitigating fibroblast senescence. Based on these mechanistic findings, we constructed a targeted delivery system by loading Rho into mesoporous silica nanoparticles and coating them with macrophage membranes (M‐Rho). The M‐Rho were then incorporated into a poly(L‐lysine)‐grafted hyperbranched poly(amidoamine) (PLL‐*g*‐HPA) hydrogel to fabricate microneedles (Gel@M‐Rho MN). This integrated system enables macrophage targeting, controlled drug release, favorable mechanical properties, and excellent biocompatibility. Moreover, the system exhibits potent reactive oxygen species (ROS)‐scavenging activity and effectively inhibits the growth of *Escherichia coli* and *Staphylococcus aureus*. In vivo animal studies confirmed that Gel@M‐Rho MN significantly promotes M2 macrophage polarization, suppresses fibroblast senescence, and accelerates wound closure, angiogenesis, and collagen deposition. Collectively, this strategy, which modulates macrophage‐fibroblast crosstalk, offers a multi‐mechanistic synergistic therapeutic approach for diabetic wounds, holding substantial potential for clinical translation.

## Graphical abstract

Diabetic wound healing is hindered by a pathological crosstalk wherein elevated IL‐11 induces M1 macrophage polarization, leading to IL‐1β/IL‐6 release and subsequent fibroblast senescence via RAS/p38MAPK/p53/E2F1 signaling. Here, we develop a Gel@M‐Rho microneedle patch that delivers rhoifolin‐loaded, macrophage membrane‐coated nanoparticles to target and reprogram macrophages. This system inhibits M1 polarization, promotes choline secretion, and alleviates fibroblast senescence, thereby breaking the vicious cycle. In diabetic mice and beagles, the patch accelerates wound closure, promotes angiogenesis, and enhances collagen deposition, offering a promising immunomodulatory strategy for diabetic wound repair.

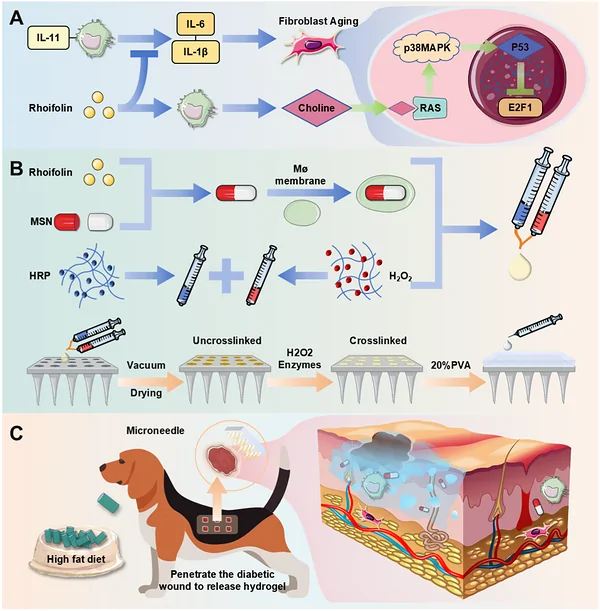

## 1 Introduction

The rising global prevalence of diabetes has rendered diabetic wounds a formidable challenge in regenerative medicine, owing to their complex pathological mechanisms and substantial socioeconomic burdens [1]. Unlike acute wounds, the diabetic wound microenvironment is characterized by persistent inflammation, oxidative stress, and premature cellular senescence, which mutually reinforce each other both spatially and temporally, forming a vicious cycle that impedes healing [2]. Among the numerous cells involved in tissue repair, dermal fibroblast, and macrophages play central roles: Fibroblast is responsible for extracellular matrix deposition, wound contraction, and tissue remodeling [3, 4], while macrophages coordinate the initiation, maintenance, and resolution of inflammation through a dynamic transition from a pro‐inflammatory (M1) to a pro‐reparative (M2) phenotyp [5]. However, in the diabetic state, this phenotypic switch is frequently impaired with M1 macrophages remaining persistently dominant, thereby leading to healing stagnation [6].

Despite extensive research on the independent functions of these two cell types, the crosstalk mechanisms between them during skin repair remain poorly understood. Recent studies have begun to unveil the existence and disruption of this crosstalk. For instance, CD301b‐expressing macrophages activate the proliferation of myofibroblast subsets, particularly adipocyte precursors, via insulin‐like growth factor 1 and platelet‐derived growth factor C [7]. Another study demonstrated that in aged skin wounds, a pro‐inflammatory/glycolytic Arg1+ macrophage subset is significantly increased, and this subset exhibits a higher potential to signal to Fibroblast through interleukin‐1 (IL‐1) compared to young skin [8]. Although these findings confirm the existence of pathological crosstalk, the upstream initiating signals and core molecular mediators remain largely undefined, particularly regarding their specificity in the diabetic wound healing process.

In our preliminary observations, we found that interleukin‐11 (IL‐11) was significantly elevated in diabetic wound tissues, and its levels were closely correlated with enhanced M1 macrophage polarization, increased expression of the senescence marker p21, and decreased expression of the proliferation marker PCNA in Fibroblast. IL‐11, a member of the IL‐6 family, has recently been redefined as a “master regulator” of tissue fibrosis and inflammatory senescence [9]. A landmark study published in Nature in 2024 revealed that IL‐11 can drive the transformation of Fibroblast into myoFibroblast independently of the TGF‐β signaling pathway and form an “IL‐11 autocrine loop” that accelerates fibrotic progression [10]. Additionally, a 2025 study showed that IL‐11 directly induces senescence in pulmonary Fibroblast and epithelial cells by activating the JAK2/STAT3 and MEK/ERK pathways, acting as a key driver of “inflammaging” [11]. However, most of these studies have focused on fibrotic diseases or normal aging. Whether IL‐11 specifically drives pathological M1 polarization of macrophages in diabetic wounds, and subsequently exacerbates dermal fibroblast senescence via paracrine signaling, represents a critical gap in our understanding of this upstream immune‐stromal crosstalk axis.

Given that IL‐11 is increasingly recognized as an attractive therapeutic target, its clinical translation has begun to emerge. For example, a dual‐network hydrogel dressing loaded with IL‐11‐targeting siRNA was developed to achieve controlled local release during the mid‐to‐late stages of wound healing, effectively inhibiting myofibroblast activation [12]. However, existing strategies mostly focus on directly neutralizing IL‐11 or silencing its gene, and have not yet approached intervention from the perspective of “modulating macrophage phenotypic remodeling and metabolic product release.” The flavonoid glycoside Rho (Rho) possesses well‐defined anti‐inflammatory and immunomodulatory activities. Our preliminary studies found that Rho effectively inhibits IL‐11‐induced M1 polarization and promotes the release of a protective metabolite—choline—from macrophages. Recent studies have confirmed that choline‐functionalized biohydrogels can create a pro‐reparative microenvironment by scavenging reactive oxygen species and inducing M2 polarization [13]. These findings provide strong evidence supporting our proposed novel signaling axis: “Rhoifolin—macrophage‐derived choline release—inhibition of fibroblast senescence.”

However, precisely delivering Rho to target cells in the deep layers of the wound, achieving long‐term and controlled release, and concurrently overcoming oxidative stress and infection within the wound microenvironment remain significant technical bottlenecks. In recent years, macrophage membrane biomimetic technology has demonstrated unique advantages for active targeting of inflammatory lesions. For instance, Mac@DM‐MOF microneedles accelerate diabetic wound healing by activating the SPP1‐ApoE axis to simultaneously induce M2 macrophage polarization and endothelial angiogenesis [14]. Another study loaded macrophage membrane‐coated kinsenoside into hydrogel microneedles to accelerate wound healing through metabolic reprogramming [15]. Inspired by these advances, we innovatively encapsulated Rho in mesoporous silica nanoparticles and coated them with macrophage membranes (M‐Rho), which were then integrated into a PLL‐*g*‐HPA hydrogel microneedle system (Gel@M‐Rho MN) possessing both antioxidant and antibacterial properties.

Collectively, we propose the following scientific hypothesis: Elevated IL‐11 in diabetic wounds drives M1 macrophage polarization, which activates inflammatory pathways such as TNF‐α to release IL‐1β and IL‐6, thereby inducing dermal fibroblast senescence and hindering healing. The Gel@M‐Rho MN system, through active macrophage targeting via the membrane coating, skin barrier penetration by the microneedles, and controlled release from the hydrogel, can precisely deliver Rho to the wound site. This reverses M1 polarization while simultaneously promoting the release of the protective metabolite choline, thereby inhibiting the downstream RAS/p38MAPK/p53/E2F1 senescence signaling axis and systematically promoting diabetic wound healing (Scheme 1). This study not only reveals, for the first time, the novel function of IL‐11 as a key mediator of pathological macrophage‐fibroblast crosstalk, but also provides both theoretical and experimental evidence for developing composite biomaterials that integrate immunomodulation and metabolic reprogramming, offering a promising strategy with enhanced clinical translational potential for the treatment of refractory diabetic wounds.

**SCHEME 1:**
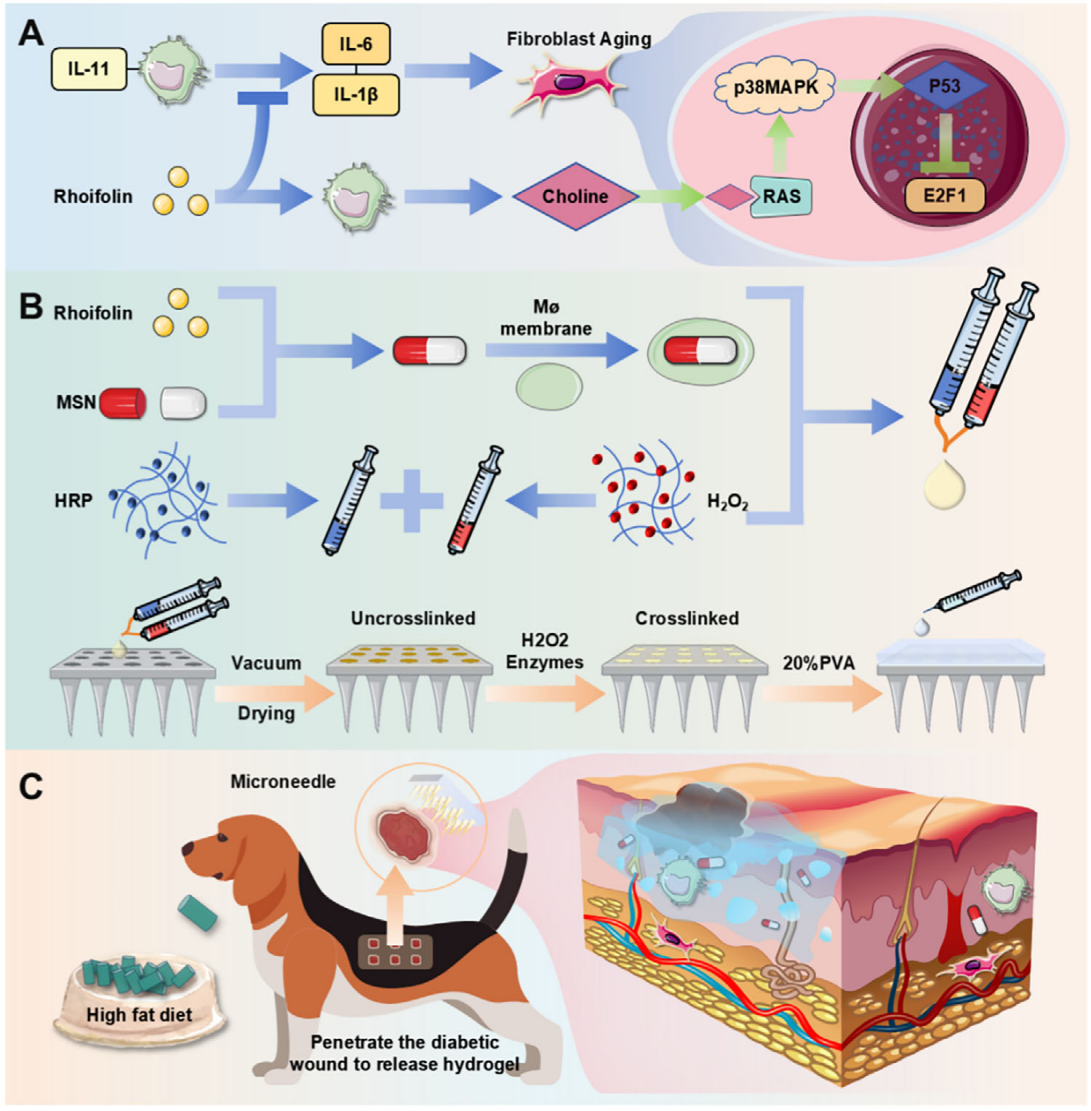
Preparation of Gel@M‐Rho microneedle (Gel@M‐Rho MN) and its mechanism in promoting the healing of diabetic wounds. (A) Rhoifolin alleviates fibroblast senescence through dual mechanisms. (B) Synthesis of Gel@M‐Rho MN. (C) Gel@M‐Rho MN action on diabetic wounds in beagles.

## 2 Results and Discussion

### 2.1 IL‐11 Increases, Macrophages Polarize Toward M1 Type, and Dermal Fibroblast’ Proliferation Diminishes in Diabetic Wounds

To elucidate the phenotypic alterations of immune and stromal cell in diabetic wound tissue, we first compared skin samples from diabetic foot ulcer patients with those from acute trauma patients. To assess macrophage polarization status, immunohistochemical staining was performed to detect the M1 marker CD86 and the M2 marker CD206. The results revealed a significant accumulation of CD86^+^ macrophage and a marked reduction of CD206^+^ macrophage in diabetic wounds (Figure 1A). To screen for aberrantly expressed inflammatory factors, immunofluorescence staining was employed, demonstrating that IL‐11 was significantly elevated in diabetic wound tissue compared to acute wounds (Figure 1B). Meanwhile, to evaluate the proliferative activity of wound cells, the expression of the proliferation marker PCNA was examined. The results showed a pronounced decrease in PCNA levels within the diabetic wound bed (Figure 1B). To determine whether this proliferation inhibition originated from fibroblast senescence, double immunofluorescence staining for the senescence‐associated protein p21 and the fibroblast‐specific marker Vimentin was performed. The results indicated that p21^+^Vimentin^+^ fibroblast was significantly increased in diabetic wounds compared to the acute control group, suggesting impaired proliferative capacity of dermal fibroblast (Figure 1C). To verify the expression changes of senescence‐ and inflammation‐related molecules at the transcriptional level, quantitative RT‐PCR was first employed to detect the mRNA expression of corresponding genes. The results showed that the transcription levels of the senescence‐associated genes *Trp53* and *Cdkn2a*, as well as the inflammatory cytokines *Il1b* and *Il6*, were all significantly upregulated (Figure 1D). To further confirm these changes at the protein level, Western blotting was performed on lysates of diabetic skin tissues. The results revealed elevated expression of the senescence‐associated proteins p53 and p16, along with a significant increase in the inflammatory cytokines IL‐1β and IL‐6, which were consistent with the transcriptional changes (Figure 1E).

**FIGURE 1:**
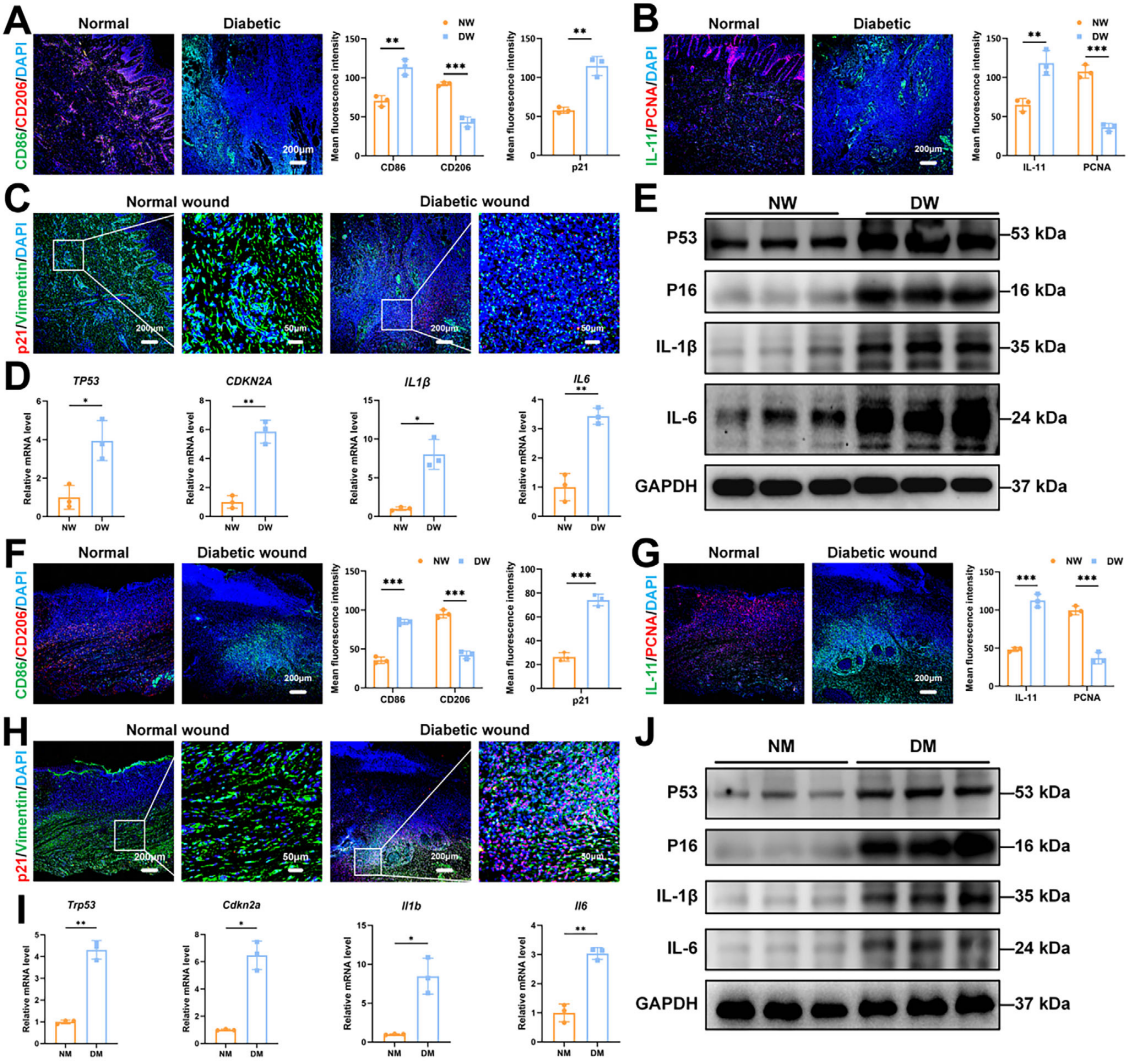
Macrophage M1‐type polarization and fibroblast senescence in diabetic wounds. (A) CD86/CD206 and (B) IL‐11/PCNA immunofluorescence images and quantitative analysis of mean fluorescence intensity. (C) p21/Vimentin immunofluorescence images and quantitative analysis of mean fluorescence intensity. (D) qPCR detection results of cell senescence (TP53, CDKN2A) and inflammation related mRNA (IL1β, IL6), and (E) western blot of related proteins. (F) CD86/CD206 and (G) IL‐11/PCNA immunofluorescence images and quantitative analysis of mean fluorescence intensity. (H) p21/Vimentin immunofluorescence images and quantitative analysis of mean fluorescence intensity. (I) qPCR detection results of cell senescence (Trp53, Cdkn2A) and inflammation (Il1β, Il6) related mRNA, and (J) Western blot of related proteins. Each group has three human samples or mice samples. All results are expressed as mean ± SD, *n* = 3. ns, non‐significant, * *p* < 0.05, **p < 0.01, ***p < 0.001.

To validate the above clinical observations in vivo, we established full‐thickness skin excision wound models (1 cm in diameter) in diabetic mice and normoglycemic control mice. The results showed that the CD86^+^ cell population increased while the CD206^+^ cell population decreased in diabetic wounds, confirming a significant shift in the M1/M2 macrophage ratio (Figure 1F). Additionally, IL‐11 levels were elevated and the expression of the proliferation marker PCNA was reduced in diabetic wound tissues (Figure 1G). To assess the senescence status of fibroblast in mouse wounds, we performed co‐immunofluorescence staining for p21 and Vimentin on wound sections. The results showed increased p21 expression in fibroblast from diabetic mouse wounds (Figure 1H). To verify the expression trends of senescence‐ and inflammation‐related molecules at the transcriptional level, we first analyzed the mRNA expression of corresponding genes using quantitative RT‐PCR. The results showed that the transcription levels of *Trp53, Cdkn2a*, as well as *Il1b* and *Il6*, were all significantly upregulated (Figure 1I). To further confirm these changes at the protein level, Western blotting was performed on wound tissue lysates. The results revealed elevated expression of p53 and p16 proteins, along with enhanced production of IL‐1β and IL‐6, which were consistent with the transcriptional trends (Figure 1J).

Collectively, these results indicate that diabetic wounds are characterized by elevated IL‐11, M1 macrophage polarization bias, and aggravated fibroblast senescence. Therefore, we speculate that IL‐11 serves as a key upstream mediator driving M1 macrophage polarization, which further exacerbates fibroblast senescence through paracrine inflammatory signals, ultimately leading to impaired diabetic wound healing.

### 2.2 IL‐11 Induces Macrophage M1 Polarization via the IL‐11/IL‐11ra1 Axis and Promotes Fibroblast Senescence by Affecting the Release of IL‐1β and IL‐6

To further investigate the role of IL‐11 in diabetic wound healing, in vitro cell experiments were conducted. To determine whether IL‐11 directly drives macrophage M1 polarization through its receptor IL‐11rα1, Bone marrow‐derived macrophage (BMDM) was treated with IL‐11 alone or in combination with small interfering RNA targeting IL‐11rα1 (siIl11ra1). Flow cytometry analysis revealed that IL‐11 stimulation significantly increased the proportion of CD86^+^ M1 macrophage, while knockdown of siIl11ra1 markedly reversed this effect (Figure 2A). Immunofluorescence staining further corroborated these results, showing that IL‐11 upregulated the M1 marker iNOS and downregulated the M2 marker Arg‐1, whereas siIl11ra1 treatment restored the expression levels of both markers to near baseline levels (Figure 2B). To evaluate the impact of the IL‐11/IL‐11rα1 signaling axis on inflammatory cytokine production, the expression of IL‐1β and IL‐6 in BMDM under different treatments was examined. Western blot analysis demonstrated that IL‐11 stimulation significantly increased the protein levels of IL‐1β and IL‐6, while siIl11ra1 knockdown suppressed their expression (Figure 2C). To quantitatively detect the secretion levels of IL‐1β and IL‐6 in cell culture supernatants, standard curves for ELISA detection of both cytokines were established. Using IL‐1β and IL‐6 ELISA kits, the absorbance values (OD450) of serial concentrations of standards were measured via a microplate reader and concentration‐absorbance standard curves were plotted (Figure S1). The results indicated that the absorbance values of both cytokines exhibited good linearity within the detection range (R^2^ > 0.99), confirming the feasibility of quantitative ELISA detection. Based on these standard curves, ELISA was performed on the culture supernatants of BMDM from each group. The results showed that IL‐11 stimulation significantly enhanced the secretion of IL‐1β and IL‐6, while this effect was attenuated by siIl11ra1 knockdown (Figure 2D). Collectively, these results indicate that IL‐11 promotes M1 macrophage polarization and inflammatory cytokine release via the IL‐11/IL‐11rα1 axis.

**FIGURE 2:**
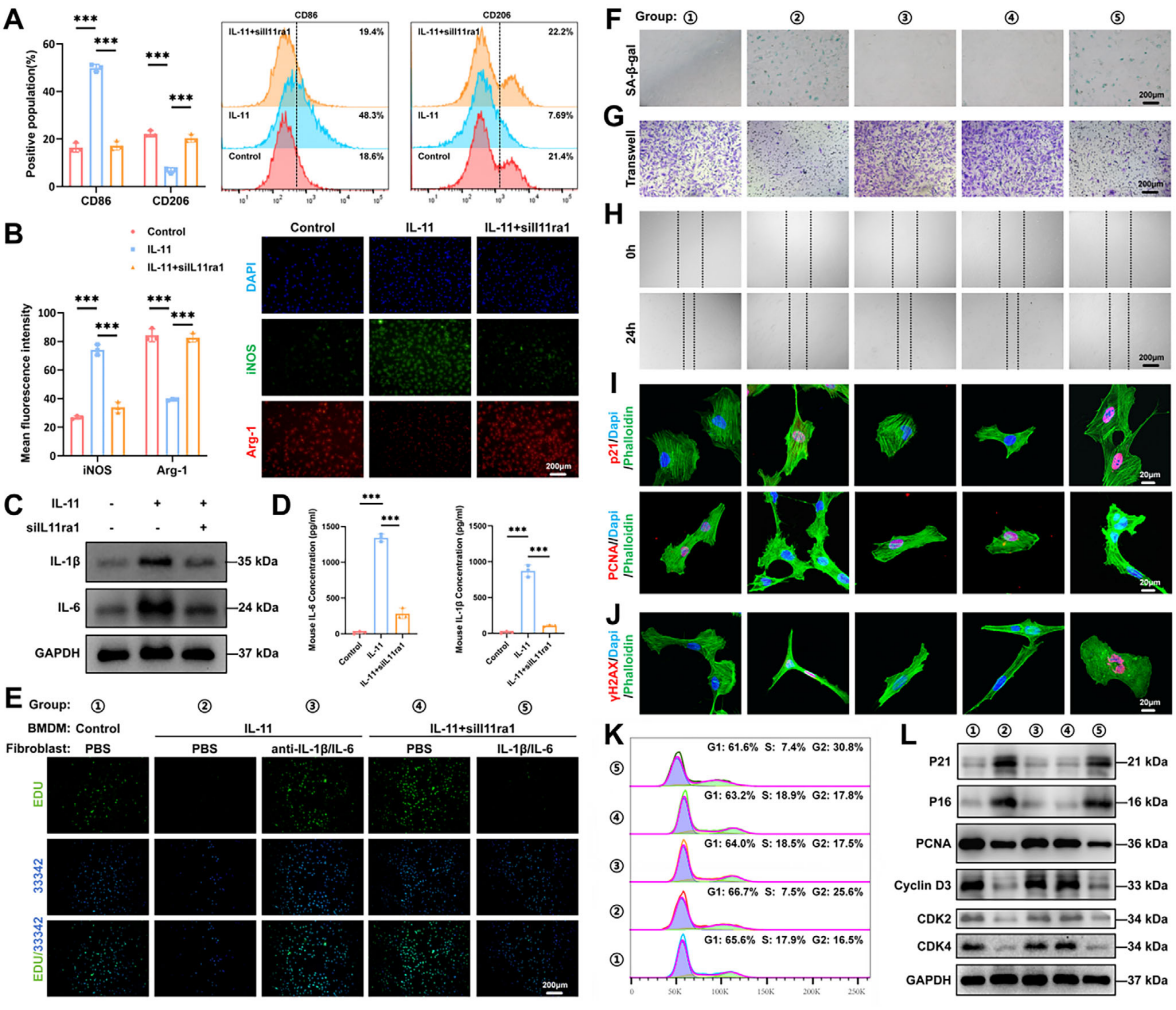
IL‐11 induces M1 macrophage polarization via the IL‐11/IL‐11rα1 axis and promotes fibroblast senescence through IL‐1β and IL‐6 release. (A) Flow cytometric analysis of M1 (CD86^+^) and M2 (CD206^+^) macrophage polarization in BMDM treated with phosphate‐buffered saline (PBS), IL‐11 alone or IL‐11 in combination with si‐Il11ra1, with quantitative analysis of the percentage of positive cells. (B) Immunofluorescence staining of iNOS and Arg‐1 in BMDM across the indicated treatment groups, with quantitative analysis of mean fluorescence intensity. (C) Western blot analysis of IL‐1β and IL‐6 protein levels in BMDM lysates and (D) ELISA quantification of IL‐1β and IL‐6 secretion in culture supernatants across the indicated treatment groups. (E) EdU proliferation assay, (F) SA‐β‐gal staining, (G) Transwell migration assay, and (H) scratch wound healing assay in fibroblast co‐cultured with BMDM subjected to the indicated interventions: Control, IL‐11, IL‐11 + anti‐IL‐1β/IL‐6, IL‐11 + siIl11ra1, and IL‐11 + siIl11ra1 + IL‐1β/IL‐6 rescue. Immunofluorescence staining of (I) p21/Phalloidin and PCNA/Phalloidin, and (J) γH2AX in fibroblast across the indicated co‐culture groups. (K) Flow cytometric cell cycle analysis and (L) Western blot analysis of proliferation‐associated proteins (PCNA, CyclinD3, CDK2, and CDK4) and senescence markers (p21 and p16) in fibroblast following co‐culture with BMDM under the indicated conditions. All results are expressed as mean ± SD, *n* = 3. ns, non‐significant, * p < 0.05, **p < 0.01, ***p < 0.001.

To investigate whether IL‐11‐polarized macrophage affects fibroblast senescence through paracrine effects, the primary dermal fibroblast used in the experiments was first identified. Observation under an inverted phase‐contrast microscope revealed that the isolated cell exhibited a typical elongated or star‐shaped morphology, arranged in a swirling pattern at high density, consistent with the morphological characteristics of fibroblast (Figure S2A). To further confirm cell purity, immunofluorescence staining was performed to detect the fibroblast‐specific markers vimentin and fibronectin. The results showed that the isolated cell was vimentin^+^ fibronectin^+^ double‐positive (Figure S2B), confirming that the obtained cell was indeed dermal fibroblast suitable for subsequent experiments. Subsequently, a Transwell co‐culture system was established (Figure S2C), with BMDM seeded in the upper chamber (4 µm pore size) and dermal fibroblast in the lower chamber. Fibroblast was separately treated with conditioned media from BMDM subjected to different treatments: Control, IL‐11, IL‐11 plus IL‐1β, and IL‐6 neutralizing antibodies (IL‐11+anti‐IL‐1β/IL‐6), IL‐11 combined with siIl11ra1 knockdown, and IL‐11 combined with siIl11ra1 knockdown plus exogenous IL‐1β and IL‐6 supplementation (IL‐11+siIl11ra1+IL‐1β/IL‐6). The EdU incorporation assay showed that conditioned medium from IL‐11‐treated BMDM significantly reduced the percentage of EdU‐positive fibroblast, indicating inhibited proliferative capacity. This effect was reversed by IL‐1β/IL‐6 neutralizing antibodies or siIl11ra1 knockdown, while the rescue experiment (adding IL‐1β and IL‐6 to the supernatant of the siIl11ra1 knockdown group) restored the inhibitory effect, confirming that IL‐1β and IL‐6 are key paracrine factors mediating fibroblast suppression (Figure 2E). Senescence‐associated β‐galactosidase (SA‐β‐gal) staining exhibited a similar trend: Fibroblast exposed to supernatant from IL‐11‐treated macrophage showed an increased SA‐β‐gal positive rate, which was alleviated by IL‐1β/IL‐6 neutralizing antibodies or si‐Il11ra1 knockdown, and reappeared after IL‐1β/IL‐6 supplementation (Figure 2F). Transwell migration and scratch wound healing assays further demonstrated that supernatant from IL‐11‐treated macrophage significantly impaired fibroblast migration capacity. This effect was partially reversed by IL‐1β/IL‐6 neutralizing antibodies or siIl11ra1 knockdown, and was re‐suppressed after IL‐1β/IL‐6 supplementation (Figure 2G, H). Immunofluorescence staining of fibroblast for p21 and phalloidin showed that supernatant from IL‐11‐treated macrophage increased p21 expression while decreasing PCNA expression; these effects were reversed by IL‐1β/IL‐6 neutralizing antibodies or siIl11ra1 knockdown, and restored after IL‐1β/IL‐6 supplementation (Figure 2I). To further evaluate DNA damage and cell cycle progression, γH2AX staining and flow cytometric cell cycle analysis were performed. Supernatant from IL‐11‐treated macrophage induced a significant increase in γH2AX foci, indicating the occurrence of DNA damage. This effect was attenuated by IL‐1β/IL‐6 neutralizing antibodies or siIl11ra1 knockdown and re‐induced after IL‐1β/IL‐6 supplementation (Figure 2J). Cell cycle analysis showed that fibroblasts exposed to IL‐11‐treated macrophage supernatants exhibited a significant reduction in the proportion of S phase (17.9% to 7.5%) and accumulation of G2/M phase cells compared with the control group. This is a classic hallmark of cell cycle arrest at the G2/M checkpoint [16]. This effect was reversed by IL‐1β/IL‐6 neutralizing antibodies or si‐Il11ra1 knockdown (S phase restored to 18.5% and 18.9%, respectively) and reappeared after IL‐1β/IL‐6 supplementation (S phase ratio decreased to 7.4%), confirming the cell cycle dysregulation induced by IL‐1β and IL‐6 in Fibroblasts (Figure 2K). S‐phase arrest is a hallmark of replication stress and cellular senescence, indicating impaired DNA synthesis and cell cycle progression [16]. Consistently, Western blot analysis demonstrated that supernatant from IL‐11‐treated macrophage downregulated the proliferation‐associated proteins PCNA, CyclinD3, CDK2, and CDK4, while upregulating the senescence markers p21 and p16. These changes were counteracted by IL‐1β/IL‐6 neutralizing antibodies or si‐Il11ra1 knockdown and re‐induced after IL‐1β/IL‐6 supplementation, confirming that IL‐11‐polarized macrophage promotes fibroblast senescence through IL‐1β‐ and IL‐6‐dependent paracrine signaling (Figure 2L).

Collectively, these results demonstrate that IL‐11 drives M1 macrophage polarization and subsequent IL‐1β and IL‐6 release through the IL‐11/IL‐11rα1 axis. These cytokines, acting as key paracrine mediators, induce fibroblast senescence, DNA damage, cell cycle arrest, and impaired proliferation and migration capacity.

### 2.3 Rhoifolin Alleviates Fibroblast Senescence by Inhibiting M1 Polarization of Macrophages and Reducing the Release of IL‐1β and IL‐6

Rhoifolin (Rho) is a natural flavonoid compound extracted from citrus fruits and the traditional Chinese medicine Exocarpium Citri Grandis (Huajuhong) [17], and has been reported to possess significant anti‐inflammatory and antioxidant activities. For instance, in animal models, it protects against cecal ligation and puncture‐induced sepsis by modulating inflammatory pathways [18], and regulates oxidative stress and pro‐inflammatory cytokine levels in Freund's adjuvant‐induced rheumatoid arthritis [19]. Although Rho exhibits anti‐inflammatory potential in classical LPS models, its fine‐tuned regulation of macrophage M1/M2 polarization balance under IL‐11‐specific microenvironment, as well as its underlying molecular networks, remain largely unknown. To determine the optimal concentration of Rho, BMDM was treated with gradient concentrations of Rho (0, 5, 10, 20, 40, 80, and 160 µM), and cell viability was assessed via the CCK‐8 assay. The corresponding BMDM survival curve revealed that cell viability began to decline significantly when the drug concentration exceeded 40 µM (Figure S3A). Based on this, the optimal drug concentration range was determined to be 0 to 40 µM. To achieve the optimal effect of Rho in promoting BMDM polarization, IL‐11‐stimulated BMDM was treated with Rho at four concentrations (0, 5, 10, 20, and 40 µM). The results indicated that when the concentration exceeded 20 µM, the effect of promoting macrophage M2 polarization no longer increased significantly. Considering that the survival rate of BMDM at 20 µM was higher than that at 40 µM, the Rho concentration of 20 µM was selected for subsequent experiments (Figure S3B).

To elucidate the specific regulatory effects of Rho on macrophage inflammatory responses, BMDM was used to establish an in vitro inflammatory model. Flow cytometry results showed that, compared with the control group, IL‐11 stimulation significantly upregulated the proportion of M1 macrophage surface marker CD86‐positive cell, while downregulating the expression of the M2 marker CD206. However, Rho intervention significantly reversed the IL‐11‐induced M1 polarization trend, manifested as decreased CD86 expression and increased CD206 expression (Figure 3A). Immunofluorescence staining further confirmed this finding, showing that the mean fluorescence intensity of iNOS in the IL‐11+Rho group was significantly reduced compared with the IL‐11 alone group, while the fluorescence signal of Arg‐1 was notably restored (Figure 3B). Furthermore, Western blot and ELISA results consistently demonstrated that IL‐11 stimulation significantly promoted the intracellular protein expression and supernatant secretion of IL‐1β and IL‐6, while co‐treatment with Rho effectively downregulated the levels of these two key pro‐inflammatory cytokines (Figure 3C‐D). These results suggest that Rho can significantly inhibit IL‐11‐induced macrophage M1 polarization and reduce the release of IL‐1β and IL‐6.

**FIGURE 3:**
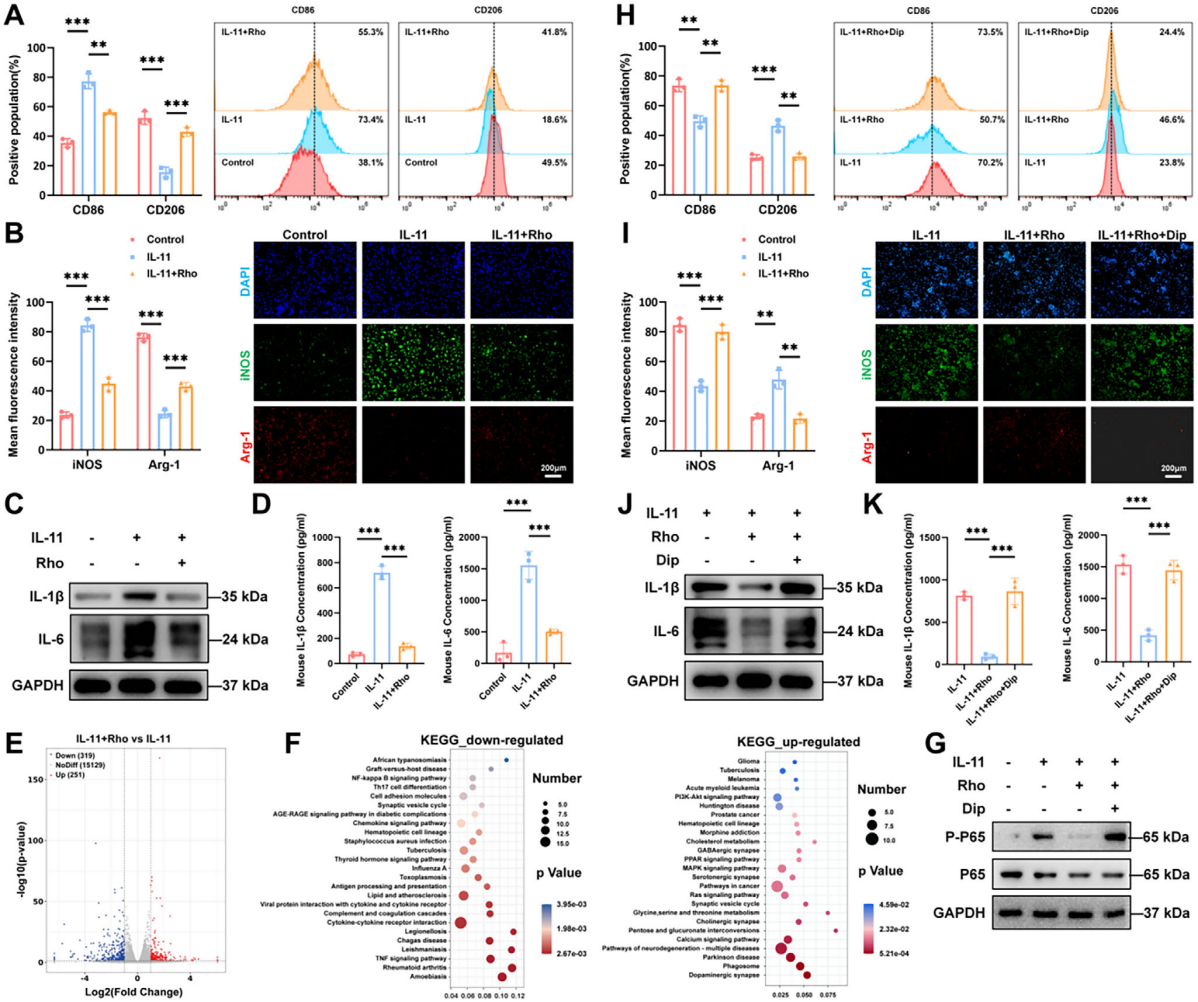
Rhoifolin inhibits M1 polarization of macrophages and reduces the release of IL‐1β and IL‐6 partly through the NF‐κB pathway. (A) Flow cytometric analysis of M1 (CD86^+^) and M2 (CD206^+^) macrophage polarization in BMDM treated with Control, IL‐11, or IL‐11+Rhoifolin. (B) Immunofluorescence staining and quantification of iNOS and Arg‐1 in BMDM under the indicated conditions. (C) Protein expression levels and (D) supernatant secretion concentrations of IL‐1β and IL‐6 in BMDM detected by Western blot and ELISA, respectively. (E) Volcano plot and (F) KEGG pathway enrichment analysis of differentially expressed genes between IL‐11 and IL‐11+Rhoifolin groups based on RNA‐seq. (G) Western blot analysis of P‐P65 and total P65 activation levels in BMDM under different treatments. H) Flow cytometry and (I) immunofluorescence staining of M1/M2 markers in BMDM after treatment with IL‐11, IL‐11+Rho, or IL‐11+Rho+Diprovocim. (J) Western blot and (K) ELISA analysis of IL‐1β and IL‐6 levels in BMDM treated with IL‐11, IL‐11+Rho, or IL‐11+Rh+Diprovocim. All results are expressed as mean ± SD, *n* = 3. ns, non‐significant, * p < 0.05, **p < 0.01, ***p < 0.001.

To further elucidate the potential molecular mechanisms by which Rho alleviates macrophage inflammatory polarization, RNA‐seq analysis was performed on the IL‐11 alone group and the IL‐11+Rho group. The volcano plot revealed that Rho induced significant alterations in the gene expression profile, with 570 differentially expressed genes identified between the two groups, including 319 downregulated and 251 upregulated genes, with excellent reproducibility among biological replicates (Figure 3E). KEGG pathway enrichment analysis indicated that these differentially expressed genes were predominantly enriched in classical inflammation‐related pathways, including the TNF signaling pathway, IL‐17 signaling pathway, and NF‐κB signaling pathway (Figure 3F). Given that NF‐κB is a core transcription factor regulating M1 polarization and the transcription of multiple pro‐inflammatory cytokines, we further examined the phosphorylation level of its key subunit p65. Western blot results showed that IL‐11 significantly induced p65 phosphorylation activation, while Rho markedly suppressed this activation process (Figure 3G), indicating that the anti‐inflammatory effect of Rho is at least partially dependent on blockade of the NF‐κB signaling pathway.

To verify the central role of the NF‐κB signaling pathway in Rho‐mediated anti‐inflammatory effects, we introduced the specific NF‐κB activator Diprovocim (Dip) [20] to conduct in vitro rescue experiments. Flow cytometry results showed that, compared with the IL‐11+Rho group, further addition of Dip re‐elevated the expression of the M1 marker CD86 and re‐suppressed the expression of the M2 marker CD206 (Figure 3H). Immunofluorescence staining also showed that iNOS fluorescence intensity rebounded while Arg‐1 fluorescence intensity decreased (Figure 3I), indicating that Dip significantly reversed the inhibitory effect of Rho on M1 polarization. Consistently, Western blot and ELISA results demonstrated that Dip addition significantly elevated the protein expression and secretion levels of IL‐1β and IL‐6 compared with the IL‐11+Rho group (Figure 3J‐K).

Given that the above results confirmed that Rho effectively inhibits IL‐11‐induced macrophage M1 polarization and pro‐inflammatory cytokine release, we further investigated the effects of macrophage secretions after Rho intervention on fibroblast biological functions. To this end, three groups were established in the fibroblast‐macrophage co‐culture system: ① IL‐11‐induced macrophage + fibroblast; ② IL‐11+Rho‐treated macrophage + fibroblast; ③ IL‐11+Rho‐treated macrophage (supplemented with exogenous IL‐1β and IL‐6) + fibroblast. First, fibroblast proliferative capacity was evaluated by the EdU proliferation assay. The results showed that co‐culture with IL‐11‐stimulated macrophage significantly inhibited the EdU‐positive rate of fibroblast; Rho intervention markedly restored its proliferative activity; however, supplementation with IL‐1β/IL‐6 on the basis of Rho intervention again decreased the EdU‐positive rate of fibroblast, reversing the protective effect of Rho (Figure S4A). SA‐β‐gal senescence staining yielded consistent results: the IL‐11 group induced a prominent senescent phenotype with blue‐stained fibroblast, the Rho group showed a significant reduction in senescent cell, while supplementation with IL‐1β/IL‐6 again increased the proportion of senescent cell (Figure S4B). In terms of functional migration, Transwell migration and scratch wound healing assays jointly confirmed that the IL‐11‐induced inflammatory microenvironment significantly suppressed fibroblast migration and repair capacity; Rho intervention markedly improved cell migration and scratch healing capacity, while supplementation with IL‐1β/IL‐6 attenuated this pro‐migratory effect of Rho, with cell migration rate and healing speed again decreased (Figure S4C‐D). At the mechanistic level, immunofluorescence staining further revealed the molecular dynamics of cell proliferation and senescence. In the IL‐11 group, fibroblast exhibited prominent proliferative arrest and senescent characteristics, with significantly enhanced p21 fluorescence signal and markedly diminished PCNA fluorescence expression; meanwhile, the expression of the DNA damage marker γH2AX was also significantly elevated. Rho intervention effectively reversed these phenotypes, significantly reducing the expression of p21 and γH2AX and restoring PCNA fluorescence signal. However, after IL‐1β/IL‐6 supplementation on the basis of Rho intervention, these protective effects were partially counteracted, with p21 and γH2AX expression again upregulated and PCNA expression correspondingly decreased (Figure S4E‐F).

Cell cycle analysis further corroborated these findings. The IL‐11‐induced inflammatory microenvironment significantly triggered S‐phase arrest in fibroblast (S‐phase cell proportion reaching 8.8%), suggesting impaired DNA synthesis and cell cycle progression. Rho intervention effectively relieved this S‐phase arrest, substantially rebounding the S‐phase cell proportion to 22.0%; however, after IL‐1β/IL‐6 supplementation, the S‐phase cell proportion reduced to 11.8%, partially reversing the alleviating effect of Rho on cell cycle impairment (Figure S4G). Western blot analysis further provided molecular‐level evidence for the above phenomena: compared with the IL‐11 group, the Rho group showed significantly elevated expression of G1/S phase transition key proteins (Cyclin D3, CDK2, CDK4) and PCNA, along with markedly reduced expression of the senescence‐associated proteins p21 and p16; while IL‐1β/IL‐6 supplementation significantly attenuated the regulatory effects of Rho on these proliferation‐ and senescence‐associated proteins (Figure S4H). Notably, the reversal magnitude of inflammatory indices in the IL‐1β/IL‐6 rescue experiment did not fully return to the level of IL‐11 stimulation alone, suggesting that Rho regulation of macrophage inflammatory polarization is not solely dependent on IL‐1β and IL‐6, and may involve the participation of other synergistic targets.

### 2.4 Rhoifolin Promotes the Release of Choline From BMDM, Which Subsequently Alleviates IL‐1β and IL‐6 Induced Fibroblast Senescence via the RAS/p38MAPK/p53/E2F1 Signaling Pathway

Given that IL‐1β and IL‐6 supplementation in the above rescue experiments did not fully restore the senescent phenotype of fibroblast to the baseline level observed with IL‐11 stimulation alone (Figure S4), we speculated that, in addition to inflammatory factors, Rho may also exert synergistic anti‐senescence effects by regulating other metabolic pathways. To this end, untargeted metabolomics sequencing was performed on the IL‐11 group and the IL‐11+Rho group.

The metabolomics volcano plot revealed that Rho intervention induced broad alterations in the metabolite profile of macrophage, with 171 differential metabolites identified (Figure 4A). The heatmap further illustrated the clustering distribution characteristics of differential metabolites among samples. Notably, among these numerous differential metabolites, the abundance of choline was significantly upregulated in the IL‐11+Rho group compared with the IL‐11 group (Figure 4B). Given that choline has been reported to possess biological functions in promoting cell viability and counteracting senescence [21, 22, 23], we speculated that choline produced by Rho‐treated macrophage may contribute to ameliorating fibroblast senescence in the microenvironment. KEGG secondary pathway classification and enrichment analysis indicated that the differential metabolites were predominantly enriched in membrane transport, carbohydrate, and amino acid metabolism pathways. These metabolic pathways are closely related to the synthesis, transmembrane transport, and subsequent transformation of choline, which serves as a key precursor for membrane phospholipids and an important methyl donor (Figure 4C). To biochemically validate the metabolomics data; we quantitatively measured the choline concentration in the co‐culture supernatant using a fluorometric choline assay kit. Briefly, a standard curve was established by plotting the net fluorescence change (Δ*F*) against known concentrations of choline standards (Figure 4D), and the absolute choline concentration of experimental samples was calculated based on this standard curve (Figure 4D). Consistent with the metabolomics results, the choline secretion level in the IL‐11+Rho group was significantly higher than that in the IL‐11 group, confirming that Rho treatment effectively promotes choline release from BMDM.

**FIGURE 4:**
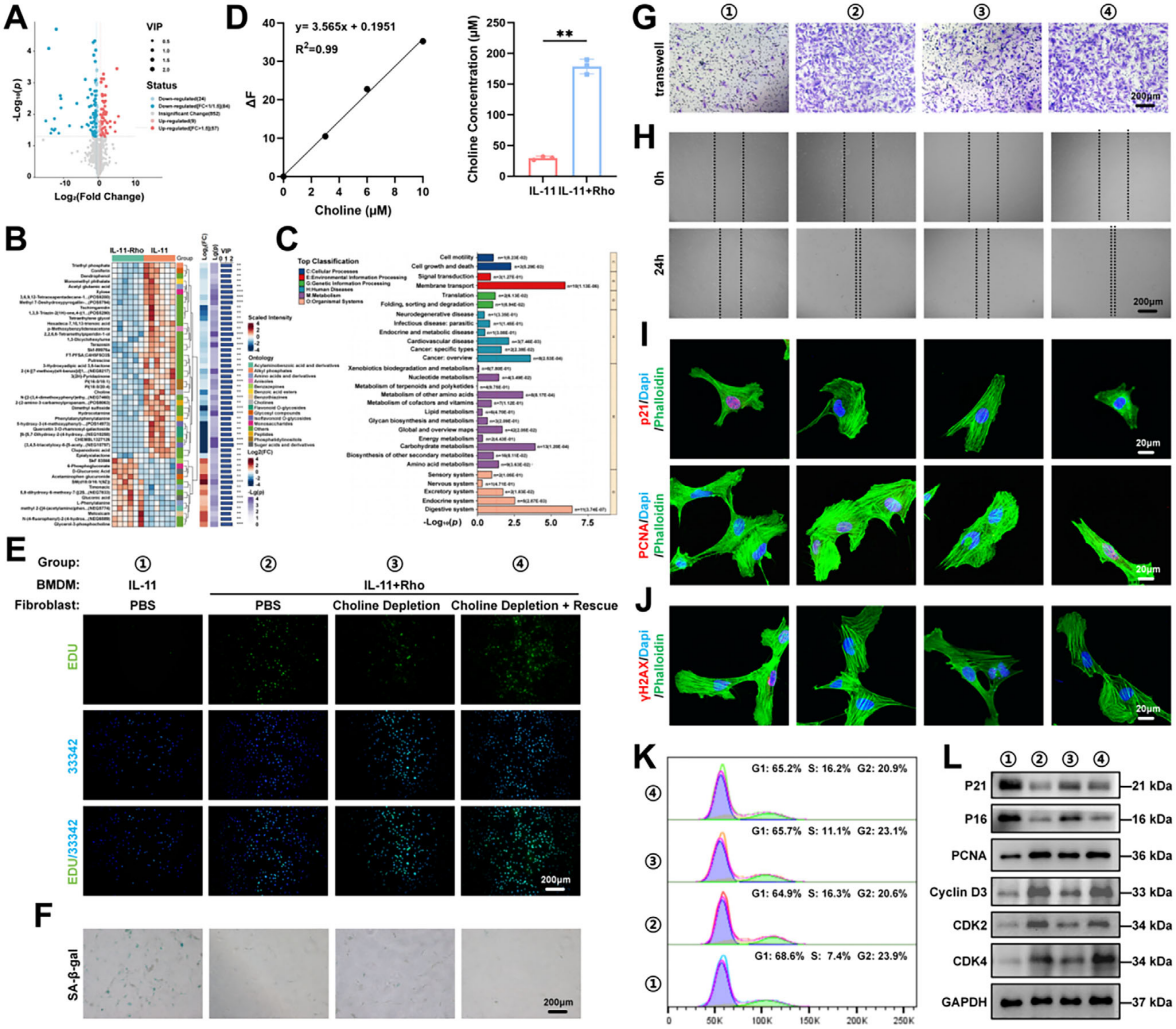
Rhoifolin inhibits fibroblast senescence by promoting choline secretion from macrophages. (A) Volcano plot and (B) hierarchical clustering heatmap of differentially abundant metabolites between IL‐11 and IL‐11+Rho groups based on metabolomics analysis. (C) KEGG secondary pathway classification of the differentially abundant metabolites. (D) Standard curve for choline quantification by plotting the fluorescence change (Δ*F*) against choline concentration and quantification of choline concentrations in the supernatants of the indicated co‐culture groups. Evaluation of fibroblast proliferation by (E) EdU incorporation assay, cellular senescence by (F) SA‐β‐gal staining, and cell migration by (G) Transwell assay and (H) wound healing assay. Immunofluorescence staining of (I) p21, PCNA, and (J) γH2AX in fibroblast. (K) Flow cytometric analysis of cell cycle distribution in fibroblast. (L) Western blot analysis of the expression levels of proliferation‐related proteins (PCNA, Cyclin D3, CDK2, CDK4) and senescence‐related proteins (p21, p16) in Fibroblast.

To verify the key role of choline in the anti‐senescence effect of Rho, rescue experiments were performed using a choline uptake/synthesis inhibitor and exogenous choline supplementation. The experiment was divided into four groups: ① IL‐11 group; ② IL‐11+Rho group; ③ IL‐11+Rho+choline inhibitor group (blocking choline action); ④ IL‐11+Rho+choline inhibitor+exogenous choline supplementation group (to verify specificity). The EdU proliferation assay showed that, compared with the IL‐11+Rho group, addition of the choline inhibitor significantly decreased the EdU‐positive rate of fibroblast, indicating that blocking choline action counteracts the pro‐proliferative effect of Rho; however, supplementation with exogenous choline on this basis restored the EdU‐positive rate (Figure 4E). SA‐β‐gal senescence staining, Transwell migration assay, and scratch wound healing assay also exhibited consistent trends: The choline inhibitor group showed aggravated senescence and reduced migration capacity, while choline supplementation partially rescued these impaired phenotypes (Figure 4F‐H). At the molecular level, immunofluorescence staining revealed that the choline inhibitor significantly reversed the downregulation of p21 and γH2AX and the upregulation of PCNA induced by Rho; after exogenous choline supplementation, the expression levels of these molecules trended back toward those of the Rho group (Figure 4I‐J). Flow cytometric cell cycle analysis showed that the S‐phase arrest proportion increased again in the choline inhibitor group, indicating that Rho's ability to alleviate S‐phase arrest was attenuated; choline supplementation restored normal cell cycle progression (Figure 4K). Western blot analysis further confirmed that after blocking choline action, the expression of proliferation‐associated proteins (Cyclin D3, CDK2, CDK4, PCNA) was significantly decreased, while the expression of senescence proteins (p21, p16) was increased; exogenous choline supplementation partially reversed these trends (Figure 4L).

Given that γH2AX immunofluorescence staining revealed that fibroblast senescence is accompanied by DNA damage (Figure 4J), and that the RAS/p38MAPK/p53/E2F1 pathway is a classical DNA damage response pathway, we hypothesized that inflammatory factors, such as IL‐1β and IL‐6 released from BMDM may promote fibroblast senescence by activating this signaling pathway, while choline may exert an inhibitory effect on this damage pathway. To verify this hypothesis, fibroblast was divided into four groups: control BMDM supernatant + normal fibroblast, IL‐11 BMDM supernatant + normal fibroblast, IL‐11 BMDM supernatant + choline group (supplemented with exogenous choline in conditioned medium), and IL‐11 BMDM supernatant + choline + choline depletor group (blocking choline action). Western blot analysis of the RAS/p38MAPK/p53/E2F1 signaling pathway revealed that IL‐11 BMDM supernatant activated this pathway in fibroblast; choline addition suppressed this signaling pathway, while the choline depletor group re‐activated the pathway, thereby validating our hypothesis (Figure S5A). To further confirm the role of this pathway, we intervened with the key molecules p38MAPK and E2F1. Four groups were established: IL‐11 BMDM supernatant + normal fibroblast, IL‐11 BMDM supernatant + choline group, IL‐11 BMDM supernatant + choline + fibroblast overexpressing p38MAPK (OE p38MAPK), and IL‐11 BMDM supernatant + choline + fibroblast with E2F1 knockdown (si E2F1). In terms of functional phenotypes, compared with the IL‐11 BMDM supernatant + normal fibroblast group, choline supplementation significantly improved fibroblast senescent phenotypes, as manifested by increased EdU proliferation, reduced SA‐β‐gal staining, restored migration capacity (Transwell and scratch assays), and relieved cell cycle arrest (Figure S5B‐E, H). However, overexpression of p38MAPK effectively reversed the protective effect of choline, leading to re‐aggravated senescence, impaired migration, and cell cycle arrest. Similarly, E2F1 knockdown also abolished the choline‐mediated anti‐senescence effect, with phenotypes returning to levels similar to those of the IL‐11 group (Figure S5B‐E, H). Immunofluorescence staining confirmed these changes: choline decreased the expression of p21 and the DNA damage marker γH2AX while increasing PCNA expression; however, both OE p38MAPK and si E2F1 significantly reversed these molecular benefits conferred by choline (Figure S5F‐G). Western blot analysis further elucidated the underlying signaling mechanisms. Choline supplementation significantly suppressed the activation of the RAS/p38MAPK/p53/E2F1 pathway induced by IL‐11 BMDM supernatant, manifested as decreased p38MAPK and p53 and increased E2F1 expression. Notably, overexpression of p38MAPK counteracted the inhibitory effect of choline and re‐activated the downstream p53/E2F1 axis; similarly, E2F1 knockdown directly blocked downstream signal transduction without affecting p38MAPK, indicating that E2F1 is the key downstream transcriptional effector in this cascade (Figure S5I). Collectively, these results indicate that choline alleviates IL‐11 supernatant‐induced fibroblast senescence by inhibiting the RAS/p38MAPK/p53/E2F1 signaling pathway.

### 2.5 Characterization of M‐Rho Nanoparticles and PLL‐*g*‐HPA Hydrogel

To achieve targeted delivery of Rho to macrophage, a macrophage membrane‐coated mesoporous silica nanoparticle system loaded with Rho (designated as M‐Rho) was constructed. The morphology of MSN and M‐Rho was observed by transmission electron microscopy (TEM). As shown in Figure 5A, the nanoparticles exhibited uniform spherical morphology before and after coating, and a distinct core‐shell structure was visible in the M‐Rho group, indicating successful membrane coating. The successful construction of M‐Rho was further verified by dynamic light scattering (DLS). As shown in Figure 5B, the average hydrodynamic diameter of MSN was 110.9 nm, which slightly increased to 119.8 nm after loading with Rho (MSN+Rho). After coating with macrophage membrane, the particle size of M‐Rho further increased to 131.4 nm, confirming successful membrane coating. Furthermore, Zeta potential measurements exhibited a distinct stepwise shift (Figure 5C). The Zeta potential of bare MSN was −45.0 mV, which shifted to −37.4 mV after drug loading, and further decreased in negative value to −25.0 mV after membrane coating. The increase in particle size accompanied by the reduction in negative charge provided strong evidence for the successful coating of MSN+Rho with macrophage membrane. SDS‐PAGE analysis revealed that the protein profile of M‐Rho was highly similar to that of purified macrophage membrane, indicating that membrane proteins were well preserved during the coating process (Figure 5D). Based on the Rho standard curve (Figure 5E), the encapsulation efficiency and drug loading capacity were determined to be 32.52 ± 2.17 wt% and 13.98 ± 0.80 wt%, respectively. Fluorescence imaging confirmed that, compared with free Rho, M‐Rho exhibited enhanced targeting ability toward BMDM and was less readily taken up by fibroblast (Figure 5F).

**FIGURE 5:**
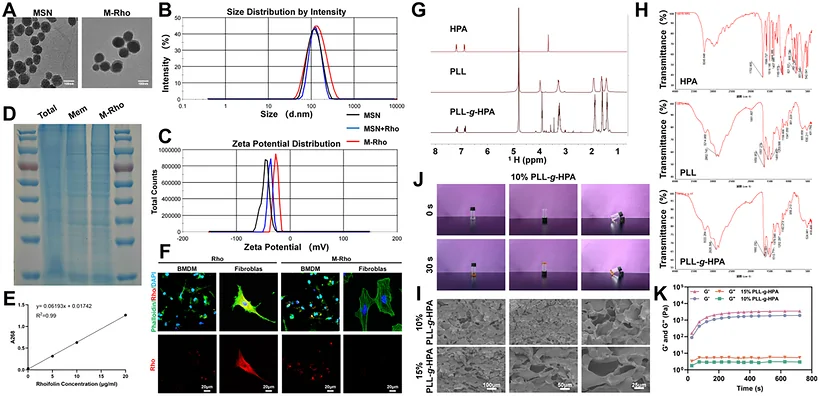
Characterization of M‐Rho nanoparticles and PLL‐*g*‐HPA hydrogel. (A) Transmission electron microscopy (TEM) images of bare MSN and M‐Rho nanoparticles. (B) Size distribution of MSN, MSN+Rho, and M‐Rho measured by dynamic light scattering (DLS). (C) Zeta potential distribution of MSN, MSN+Rho, and M‐Rho. (D) SDS‐PAGE analysis of total proteins from cell lysates (Total), BMDM membranes (Mem), and M‐Rho. (E) Standard curve of Rho for determining drug encapsulation efficiency and loading capacity. (F) Fluorescence imaging evaluating the targeting ability of free Rho and M‐Rho toward BMDM and Fibroblast. (G) ^1^H NMR spectra of HPA, PLL, and PLL‐*g*‐HPA copolymer. (H) Fourier‐transform infrared (FTIR) spectra of HPA, PLL, and PLL‐*g*‐HPA hydrogel. (I) SEM images showing the porous morphology of freeze‐dried 10% and 15% PLL‐*g*‐HPA hydrogels. (J) Photographs of the inverted tube test demonstrating the gelation process of 10% PLL‐*g*‐HPA hydrogel at 0 min and 30 s. (K) Rheological analysis of 10% and 15% PLL‐*g*‐HPA hydrogels showing storage modulus (G') and loss modulus (G'') over time.

In addition, to provide a supportive three‐dimensional microenvironment, PLL‐*g*‐HPA hydrogel was prepared. ^1^H nuclear magnetic resonance (NMR) and Fourier‐transform infrared (FTIR) spectroscopy verified the successful synthesis of the hydrogel components (Figure 5G‐H). Scanning electron microscopy (SEM) revealed that the hydrogel possessed a porous interconnected structure suitable for cell encapsulation (Figure 5I). The inverted tube test demonstrated that the hydrogel underwent rapid gelation within 30 s after mixing (Figure 5J). Rheological measurements further confirmed the viscoelastic properties of the hydrogel, with the storage modulus (G') significantly exceeding the loss modulus (G''), indicating a stable crosslinked network and favorable mechanical strength (Figure 5K). These results collectively confirmed the successful construction of the M‐Rho delivery system and the supportive hydrogel scaffold.

Furthermore, the antioxidant and antibacterial activities of PLL‐*g*‐HPA hydrogel were evaluated. To assess antioxidant capacity, BMDM was loaded with the DCFH‐DA probe to monitor intracellular total reactive oxygen species (Total ROS) levels. As shown in Figure S6A, IL‐11‐induced accumulation of total ROS in BMDM was significantly elevated. However, with increasing concentrations of PLL‐*g*‐HPA hydrogel (5%, 10%, and 15%), ROS levels were suppressed in a dose‐dependent manner, with the 10% and 15% hydrogels exhibiting superior ROS‐scavenging capacity compared with the 5% group. Meanwhile, the antibacterial properties of the hydrogel were evaluated against *E. coli* and *S. aureus* (Figure S6B). The plate colony counting assay demonstrated that both 10% and 15% PLL‐*g*‐HPA hydrogels significantly reduced the survival rates of *E. coli* and *S. aureus* compared with the blank control group (Figure S6C). SEM further revealed that the bacterial morphology in the hydrogel‐treated groups was severely disrupted, with visible membrane rupture and leakage of intracellular contents, while the bacteria in the blank group maintained intact and smooth surfaces (Figure S6D). These results confirmed that PLL‐*g*‐HPA hydrogel possesses excellent total ROS‐scavenging and antibacterial properties. Given that the ROS‐scavenging capacities of the 10% and 15% hydrogels were comparable, the 10% PLL‐*g*‐HPA hydrogel was used in subsequent experiments based on cost considerations.

### 2.6 Construction and Characterization of Gel@M‐Rho MN

To achieve efficient transdermal delivery of Rho while preserving its targeting capability, Gel@M‐Rho MN (10 × 10 array) was fabricated via a mold‐casting method, as illustrated in Figure 6A. M‐Rho nanoparticles with a drug loading content of 13.98 wt% were dispersed in the PLL‐*g*‐HPA pre‐gel solution at a concentration of 45.0 mg/mL to achieve a final concentration of 22.5 mg/mL in the 10% (w/v) PLL‐*g*‐HPA hydrogel. The mixture was injected into a 10 × 10 PDMS mold. After vacuum degassing, enzymatic crosslinking, and PVA backing casting, the final microneedle patches were obtained. Based on this formulation, each microneedle patch (100 needles per patch) contained an equivalent total Rho dose of approximately 37.75 µg (0.38 µg per needle).

**FIGURE 6:**
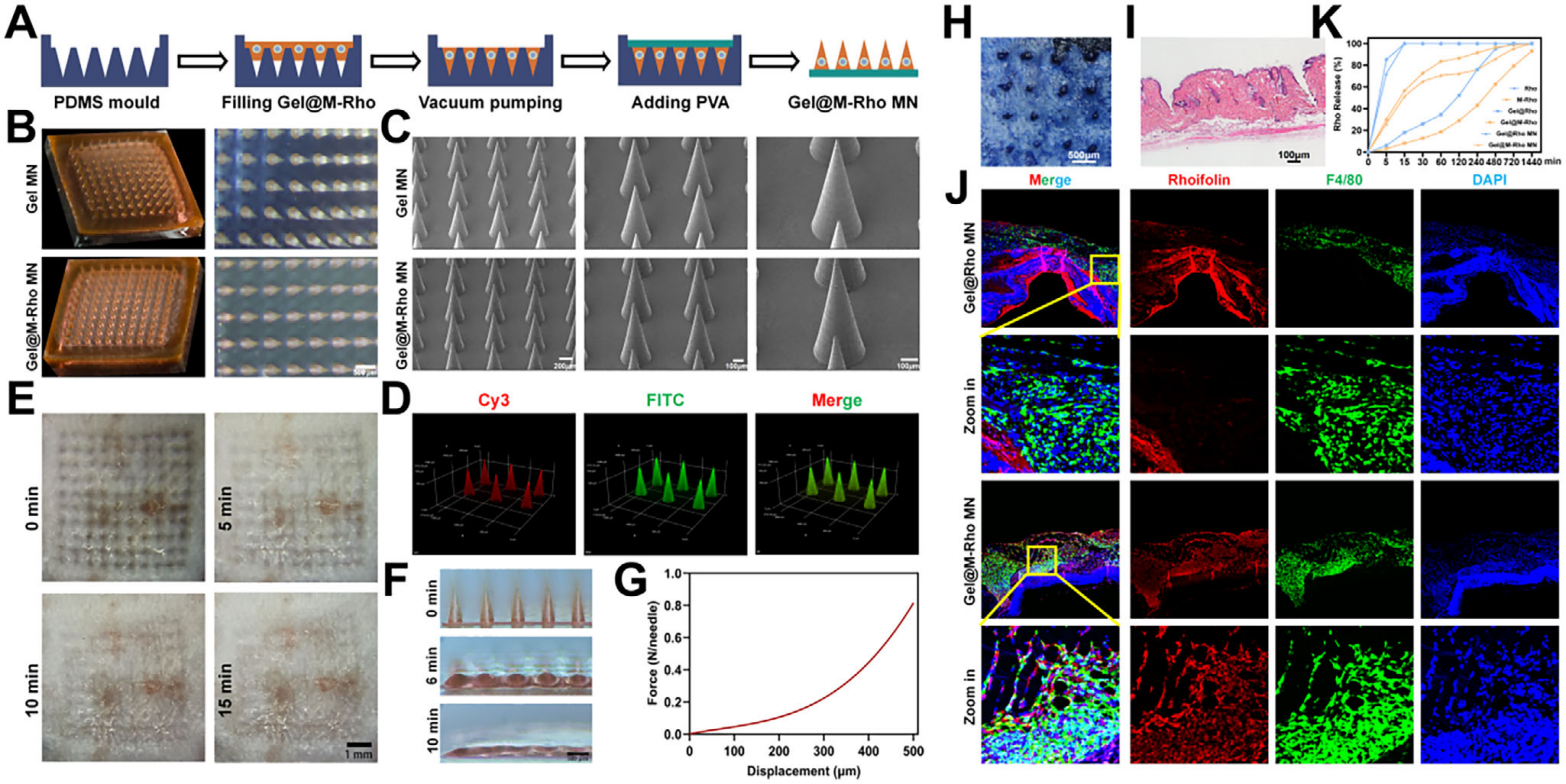
Fabrication, characterization, and release profiles of Gel@M‐Rho microneedles. (A) Schematic illustration of the fabrication process of Gel@M‐Rho MN using a PDMS mold. (B) Representative macroscopic images of Gel MN and Gel@M‐Rho MN patches observed under a stereo microscope. (C) SEM images showing the morphology and sharp tips of Gel MN and Gel@M‐Rho MN at different magnifications. (D) 3D reconstruction of confocal microscopy images showing the distribution of Cy3‐labeled M‐Rho (red) and FITC‐labeled hydrogel (green) within the microneedle tips. (E) Time‐lapse photographs of mouse dorsal skin after microneedle insertion, demonstrating the closure of micropores within 15 min. (F) Dissolution behavior of the microneedle tips in mouse skin over time (0, 6, and 10 min). (G) Force‐displacement curve of the microneedles for evaluating skin penetration capability. (H) Trypan Blue staining of the punctured skin to confirm successful transdermal penetration. (I) H&E staining of the skin section after microneedle insertion, showing the puncture depth reaching the dermis. (J) Representative immunofluorescence images showing the co‐localization of Rho (red) and macrophage marker F4/80 (green) in wound tissues treated with Gel@Rho MN and Gel@M‐Rho MN. (K) In vitro cumulative release profiles of Rhoifolin from free Rho, M‐Rho, Gel@Rho, Gel@M‐Rho, Gel@Rho MN, and Gel@M‐Rho MN over 1440 min.

Stereo microscopy and SEM revealed that both the plain hydrogel microneedles (Gel MN) and the drug‐loaded microneedles (Gel@M‐Rho MN) exhibited a well‐arranged conical array structure with intact tips (Figure 6B‐C). M‐Rho was labeled with Cy3, and the hydrogel was labeled with FITC. The microneedles were scanned under a confocal fluorescence microscope and reconstructed into three‐dimensional images, demonstrating that M‐Rho was perfectly incorporated into the hydrogel microneedles (Figure 6D). Mechanical strength testing showed that the penetration force of a single microneedle increased steadily with displacement and could withstand pressure exceeding the minimum force required for skin penetration (Figure 6G). The micropores formed on the skin surface closed spontaneously within 15 min, indicating minimal tissue trauma (Figure 6E). The in vitro dissolution test showed that the hydrogel needle tips dissolved rapidly upon hydration, gradually disappearing within 10 min and leaving only the intact PVA backing layer, indicating that the microneedles possess ideal biodegradability and can achieve rapid drug release upon skin insertion (Figure 6F). Trypan blue staining and H&E staining of skin sections confirmed its transdermal delivery capability, demonstrating that the microneedles successfully penetrated the *Stratum corneum* and delivered the drug into the dermal layer (Figure 6H‐I).

To verify the in vivo macrophage‐targeting capability of the M‐Rho system, the co‐localization of Rho (red) and the macrophage marker F4/80 (green) was examined in wound tissues. Confocal microscopy imaging revealed that Rho was significantly enriched in F4/80‐positive macrophage in the Gel@M‐Rho MN‐treated group, whereas the non‐coated formulation exhibited markedly weaker targeting (Figure 6J). Finally, the delivery kinetics of the microneedle system was evaluated via in vitro drug release assay. As shown in Figure 6K, free Rho and the microneedle loaded with drug alone exhibited a typical burst release behavior, with over 70% of the drug released within the first 5 min. In contrast, the hydrogel‐based formulations (Gel@Rho and Gel@M‐Rho) exhibited controlled release behavior due to the crosslinked polymer network serving as a diffusion barrier. Notably, the release rate of Gel@M‐Rho MN was significantly faster than that of Gel@M‐Rho, reaching approximately 70% cumulative release within 2 h. This enhanced release kinetics is primarily attributed to the dramatically increased specific surface area of the microneedle tips, which facilitates rapid fluid infiltration and polymer erosion [24]. Although the microneedle configuration sacrifices a portion of the long‐term controlled release capability compared with bulk hydrogel, this moderate acceleration is a necessary and deliberate trade‐off to achieve efficient transdermal penetration, rapid local drug accumulation, and timely therapeutic onset. Collectively, these results confirmed the successful fabrication of Gel@M‐Rho MN with excellent mechanical properties, rapid skin penetration, precise macrophage targeting, and optimized drug release kinetics suitable for diabetic wound management.

To evaluate the systemic biosafety of Gel@M‐Rho MN, a comprehensive biocompatibility assessment spanning from in vitro cytotoxicity to in vivo animal safety was performed. First, the cytotoxicity of PLL‐*g*‐HPA hydrogel toward BMDM and fibroblast was evaluated. Cells were co‐cultured with different concentrations of PLL‐*g*‐HPA hydrogel (5%, 10%, and 15%) or PBS for 24 h. As shown in Figure S7A, the CCK‐8 assay revealed that the cell survival rate in all hydrogel‐treated groups remained above 85%, with no significant difference compared with the PBS control group. Furthermore, live/dead staining confirmed that the vast majority of cells remained viable, with no obvious red (dead cell) fluorescence signal observed in any group (Figure S7B), indicating that the hydrogel material possesses excellent in vitro biocompatibility. The blood compatibility of the hydrogel was further evaluated via a hemolysis assay. As shown in Figure S7C, after incubating mouse erythrocytes with 5%, 10%, and 15% PLL‐*g*‐HPA hydrogels, the hemolysis rates were all below 5%, far lower than that of the positive control (ddH_2_O). The supernatant remained clear, indicating that the material did not induce significant erythrocyte rupture. To confirm the in vivo safety of the integrated system, Gel MN, M‐Rho, and Gel@M‐Rho MN were applied to diabetic wound mice for 14 days. Hematological analysis showed that the levels of hemoglobin (Hb), white blood cells (WBC), and platelets (PLT) in all treatment groups were comparable to those in the PBS group, indicating no hematotoxicity (Figure S7D). Similarly, serum biochemical markers, including liver function indicators (ALT and AST) and kidney function indicators (Cr and LDH), showed no significant differences among the four groups (Figure S7E), indicating that the materials did not cause hepatic or renal dysfunction. Finally, H&E staining of the major organs (heart, liver, spleen, lungs, and kidneys) was performed to examine potential organ damage. Although typical diabetic pathological features (e.g., mild hepatic steatosis) were observed in the liver sections across all groups, no obvious inflammatory infiltration, necrosis, or structural damage was observed in the heart, spleen, lungs, or kidneys (Figure S7F). Importantly, the implanted materials did not exacerbate these diabetic lesions. Collectively, these results confirmed that PLL‐*g*‐HPA hydrogel and Gel@M‐Rho MN system possess excellent biocompatibility and systemic safety, highlighting their great potential for safe clinical translation in diabetic wound therapy.

### 2.7 Gel@M‐Rho MN Promotes Diabetic Wound Healing in Mice

Based on the Gel@M‐Rho MN system, which inhibits inflammation, alleviates fibroblast senescence, and exhibits anti‐ROS and antibacterial effects, in vivo animal experiments were conducted on diabetic mice. First, C57BL/6 mice were fed a high‐fat diet continuously, followed by a 24 h fasting period and subsequent injection of streptozotocin (STZ). On day 3 post‐administration, blood glucose levels in the tail vein were monitored to confirm successful induction of the diabetic model. Subsequently, a standard wound was created on the dorsum of diabetic mice using a 1 cm diameter punch, and the mice were randomly divided into different experimental groups: control group, M‐Rho group, microneedle material group (Gel MN), and drug‐loaded microneedle group (Gel@M‐Rho MN) (Figure 7A). The wound healing process was macroscopically evaluated by capturing images on days 0, 3, 7, 10, and 14 (Figure 7B, C). The results showed significant differences among the four groups, with the Gel@M‐Rho group exhibiting the fastest healing rate, achieving near‐complete closure by the experimental endpoint. The healing efficacy of the drug alone and hydrogel microneedle material groups ranked next, while the control group showed the poorest healing outcome (Figure 7D). These results indicate that our hydrogel microneedle drug delivery system effectively promotes wound healing at the macroscopic level.

**FIGURE 7:**
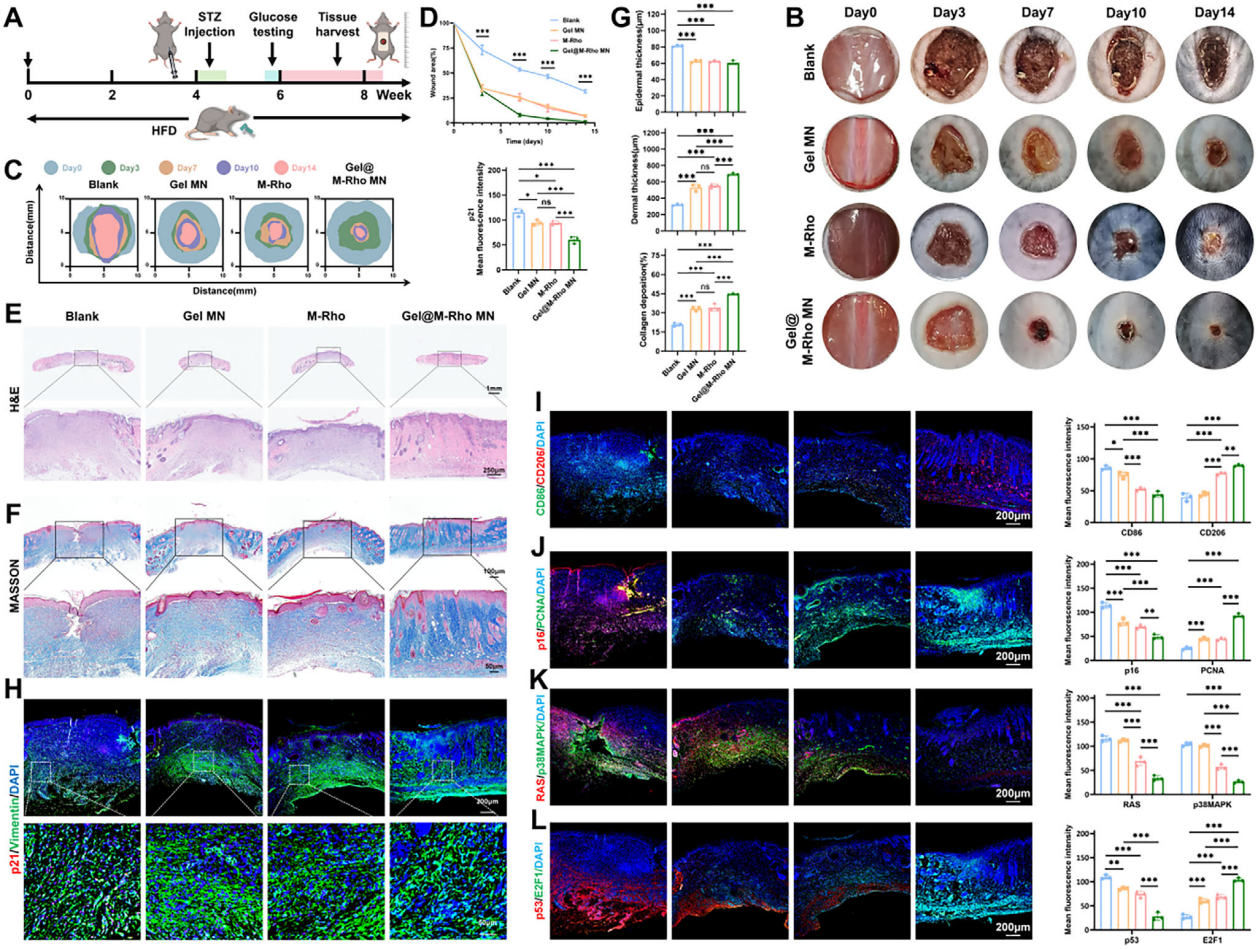
Gel@M‐Rho MN promotes diabetes wound healing in mice. (A) Schematic representation of the diabetic wound model in mice. (B) Images of wound healing appearance. (C) Schematic representation of wound healing redrawn. (D) Wound healing area statistical chart. (E) Wound H&E staining images. (F) Wound Masson's trichrome staining images. (G) The quantification of epidermal thickness, dermal thickness, and collagen volume fraction, respectively. (H) p21/vimentin immunofluorescence images and quantitative analysis of mean fluorescence intensity. (I) CD86/CD206, (J) p16/PCNA, (K) RAS/p38MAPK, and (L) p53/E2F1 immunofluorescence images and quantitative analysis of mean fluorescence intensity. Each group has three mice. ns, non‐significant, * p < 0.05; **p < 0.01; ***p < 0.001.

Meanwhile, the histological features of skin tissue repair, including collagen deposition, granulation tissue formation, and dermal thickening, were examined. In this study, histopathological measurements were consistent with macroscopic observations. Hematoxylin and eosin (H&E) staining revealed that the dermis of the Gel@M‐Rho treatment group was the thickest, surpassing that of the drug alone or microneedle alone groups, while the control group exhibited the thinnest dermis (Figure 7E, G). Furthermore, Masson's trichrome staining demonstrated that the collagen fibers within the Gel@M‐Rho group were arranged in an orderly manner, with a significantly higher volume fraction compared with the drug alone or microneedle alone groups and far exceeding the blank control group (Figure 7F, G). Therefore, these data confirmed the role of M‐Rho in diabetic wound healing and highlighted the therapeutic efficacy of microneedle material as a delivery vehicle in promoting skin tissue regeneration and enhancing collagen deposition.

Given the significant effects of the drug and material in promoting macrophage polarization toward the M2 phenotype and inhibiting fibroblast senescence, the expression of macrophage polarization markers and fibroblast senescence‐associated proteins in wound tissues from different treatment groups was evaluated. Immunohistochemistry results showed that the expression of the M2 macrophage marker CD206 was significantly enhanced in the Gel@M‐Rho treatment group, followed by the M‐Rho alone group, while the hydrogel microneedle group and the control group exhibited the weakest responses (Figure 7I). Thus, at the animal experimental level, this system effectively promoted M2 macrophage polarization. We further assessed fibroblast senescence status via immunohistochemical analysis and found that Gel@M‐Rho treatment significantly suppressed fibroblast senescence in diabetic wound skin, manifested as significantly reduced p21 expression in vimentin‐positive fibroblast compared with the M‐Rho or microneedle alone groups, with the control group exhibiting the highest expression (Figure 7H). The expression of p16 in skin tissues followed the order: blank group > M‐Rho group or microneedle group > Gel@M‐Rho group, while PCNA expression exhibited the opposite trend (Figure 7J), indicating that the antioxidant hydrogel microneedle drug delivery system effectively inhibits fibroblast senescence. We further examined the expression of fibroblast senescence pathway‐related proteins (RAS/p38MAPK/p53/E2F1) using immunohistochemistry. The expression levels of RAS, p38MAPK, and p53 followed the order: Blank group > M‐Rho group or Gel MN group > Gel@M‐Rho MN group, while E2F1 expression exhibited the opposite trend: Blank group < M‐Rho group or Gel MN group < Gel@M‐Rho group (Figure 7K, L). This further verified that the hydrogel microneedle system alleviates fibroblast senescence by inhibiting the RAS/p38MAPK/p53/E2F1 signaling pathway.

### 2.8 Gel@M‐Rho Promote Diabetes Wound Healing in Beagles

To further validate the clinical translational potential of the Gel@M‐Rho MN system in a large animal model, analogous experiments were conducted in beagles. Diabetes was induced in beagles by feeding a high‐fat diet for three consecutive months, which was confirmed by elevated blood glucose levels. A full‐thickness skin defect wound (2 cm × 2 cm) was created on the dorsal skin (Figure 8A), and images were captured on days 0, 3, 7, 10, and 14 to monitor the wound healing process (Figures 8B, 8C). As shown by the wound closure curve in Figure 8D, significant differences were observed among the four groups. The Gel@M‐Rho MN group exhibited the fastest healing rate, with the wound nearly completely closed by the experimental endpoint. In contrast, the therapeutic effects of the M‐Rho alone treatment and the Gel MN patch groups were relatively weaker, while the blank group showed the poorest healing outcome. These results indicate that our Gel@M‐Rho MN system effectively promotes diabetic wound healing at the macroscopic level in large animals.

**FIGURE 8:**
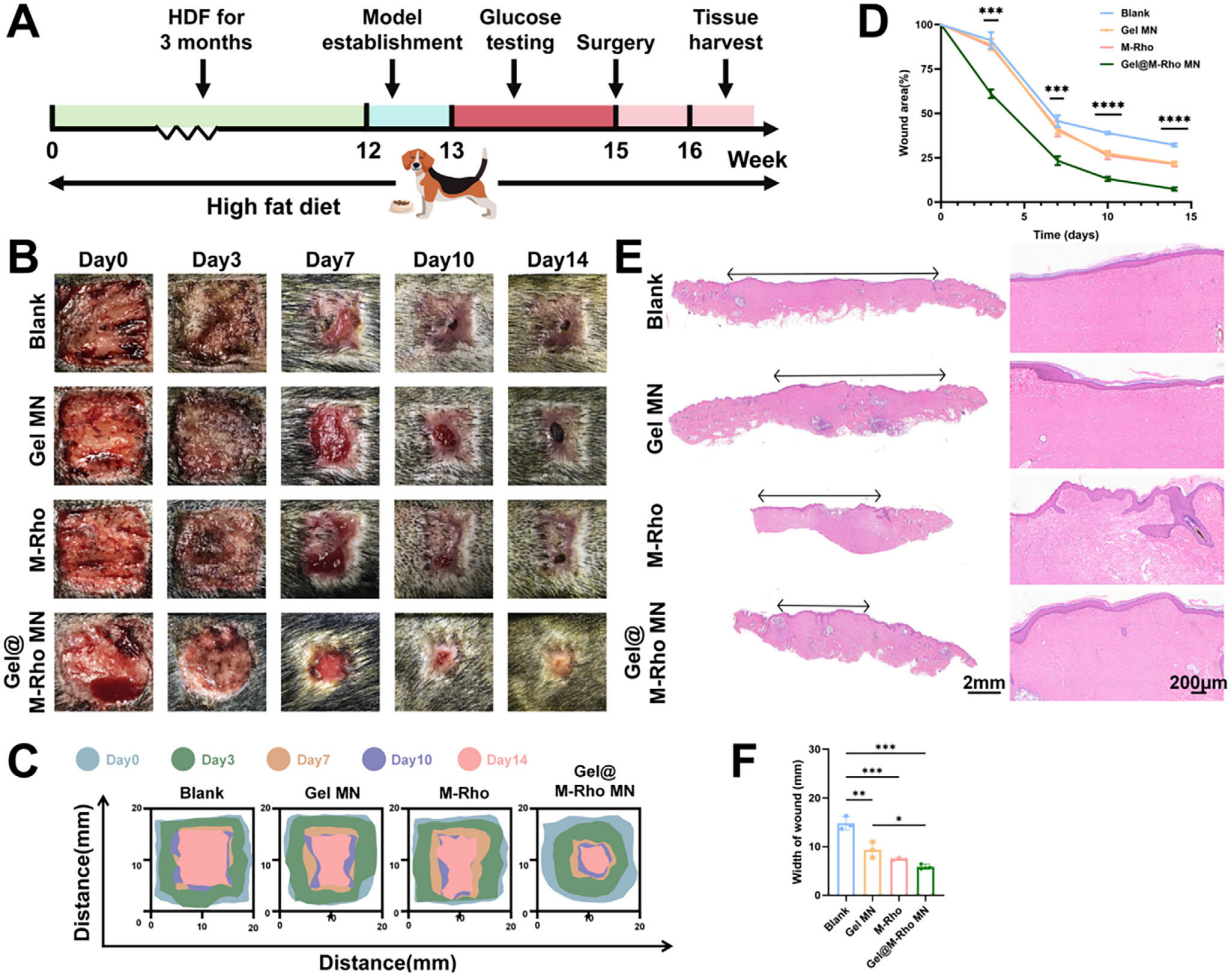
Gel@M‐Rho promote diabetes wound healing in beagle. (A) Schematic representation of diabetic wound model in beagle. (B) Images of wound healing appearance. (C) Schematic representation of wound healing redrawn. (D) Wound healing area statistical chart. (E) Wound H&E staining Images and (F) statistical chart of granulation tissue width. Each group has three beagle. ns, non‐significant, * p < 0.05, **p < 0.01, ***p < 0.001.

Histological analysis was performed by H&E staining of skin samples harvested on day 14. As shown in Figure 8E, a distinct boundary was visible between the regenerated granulation tissue and the surrounding intact muscle tissue. Quantitative analysis of wound width revealed the following trend: blank group > M‐Rho group or Gel MN group > Gel@M‐Rho MN group, indicating that the Gel@M‐Rho MN treatment group has the highest degree of wound closure and the most mature granulation tissue, effectively reducing the size of the wound (Figure 8F). These histological results were consistent with the macroscopic wound closure rates, confirming that the Gel@M‐Rho MN system accelerates the wound healing process in diabetic beagles.

## 3 Conclusion

Diabetic wound is characterized by a complex pathological microenvironment driven by persistent inflammation, oxidative stress, and cellular senescence. In this study, we elucidated a key intercellular crosstalk mechanism: elevated IL‐11 in diabetic wound induces macrophage polarization toward the M1 phenotype, which subsequently releases excessive IL‐1β and IL‐6 through activation of the TNF‐α pathway. These inflammatory mediators orchestrate fibroblast senescence via the RAS/p38MAPK/p53/E2F1 signaling cascade. Interestingly, we found that the flavonoid compound Rho effectively blocks this cascade through a dual‐action mechanism. It not only suppresses the release of IL‐1β and IL‐6 from M1 macrophage, but also promotes the secretion of the bioactive metabolite choline, which acts as a key intercellular messenger to inhibit the RAS/MAPK pathway in fibroblast.

To translate these mechanistic findings into a viable therapeutic strategy, we constructed a multifunctional microneedle patch (Gel@M‐Rho MN). By encapsulating Rho in macrophage membrane‐coated mesoporous silica nanoparticles (M‐Rho) and embedding them in a PLL‐*g*‐HPA hydrogel, this system achieves synergistic therapeutic effects: (i) Precise macrophage targeting via membrane camouflage; (ii) inherent antioxidant and antibacterial activities of the hydrogel; and (iii) controlled local transdermal delivery of Rho. This integrated system successfully reprograms the inflammatory microenvironment, alleviates fibroblast senescence, and promotes robust tissue regeneration.

The therapeutic efficacy of the Gel@M‐Rho microneedle system was comprehensively validated in diabetic mouse models, as evidenced by accelerated wound closure, enhanced collagen deposition, and effective in vivo suppression of the RAS/p38MAPK/p53/E2F1 pathway. Macroscopic pro‐healing effects were also observed in a large animal model (beagle). Collectively, this work not only provides deep insights into the metabolic crosstalk between macrophage and fibroblast in diabetic wound, but also presents a biomaterial strategy with clinical translational potential. Future efforts will focus on optimizing the mechanical properties of the microneedles to accommodate human skin thickness, and advancing the safety evaluation for clinical translation in regenerative medicine.

## 4 Experimental Section

### 4.1 Materials

Sigma: Dimethylsulfoxide‐d6 (DMSO‐d6), Triton X‐100, 40,6‐diamidino‐2‐phenylindole (DAPI), paraformaldehyde (PFA), Choline Assay Kit (MAK508).

Gibco: Phosphate buffered saline (DPBS), fetal bovine serum (FBS, #10099141), Trizol, a‐minimum essential medium (DMEM), penicillin, streptomycin, pancreatic enzyme.

Beyotime: Cytotoxicity Assay Kit, Senescence β‐Galactosidase Staining Kit, EdU Cell Proliferation Kit with Alexa Fluor 488, BCA protein assay kit, Reactive Oxygen Species Assay Kit, Cell Counting Kit‐8, Membrane protein extraction kit, Mouse IL‐1β ELISA Kit (PI301), Mouse IL‐6 ELISA Kit (PI326), Phospho‐Histone H2AX (Ser139) Rabbit Polyclonal Antibody (AF5836), PI‐Annexin V (C1052).

Thermo Fisher Scientific: RPMI 1640, 24‐well transwell system, FITC‐Phalloidin

MedChemExpress: Diprovocim, Protease Inhibitor Cocktail (EDTA‐Free, 100× in DMSO), Streptozocin, Choline chloride.

Shyuanye: Rhoifolin

Millipore: PVDF membranes

Abclonal: Protein molecular weight markers, antibodies to RAS (A23382), p38MAPK (A5049), E2F1 (A19579), p16 (A23882), p21 (A19094), PCNA (A12427), CD86 (A21198), IL‐1β (A19635), IL‐6 (A14687), Cyclin D3 (A3989), iNOS (A3774).

Proteintech: Antibodies to Vimentin (60330‐1‐Ig), Fibronectin (15613‐1‐AP), p53 (60283‐2‐Ig), ARG‐1 (66129‐1‐Ig), GAPDH (60004‐1‐Ig), IL‐11 (55169‐1‐AP), CD206 (18704‐1‐AP).

Affinity Biosciences: Antibodies to CDK2 (AF6237), CDK4 (DF6102).

Biolegend: FITC anti‐mouse/human CD11b Antibody, APC anti‐mouse F4/80 Antibody, PE anti‐mouse CD86 Antibody, PerCP anti‐mouse CD206 Antibody.

Boster: Goat serum, RIPA lysis buffer, Western blot‐specific primary and secondary antibody diluent, CY3 Conjugated AffiniPure Goat Anti‐mouse IgG (H+L), CY3 Conjugated AffiniPure Goat Anti‐rabbit IgG (H+L), FITC Conjugated AffiniPure Goat Anti‐mouse IgG (H+L), FITC Conjugated AffiniPure Goat Anti‐rabbit IgG (H+L).

### 4.2 Inclusion and Exclusion Criteria for Clinical Patients and Cohort Characteristics

Inclusion criteria for the diabetic wound group: Patients diagnosed with type 2 diabetes mellitus, presenting with full‐thickness chronic cutaneous ulcers located on the lower extremities, with a duration exceeding four weeks without healing. Age ranging from 18 to 75 years. Written informed consent was obtained prior to surgery. Complete clinical data were available, including gender, age, wound location, wound diameter, and time of injury.

#### 4.2.1 Inclusion Criteria for the Normal Acute Wound Group

Patients who underwent urgent reconstructive surgery or debridement due to acute trauma/foot injury, without any underlying systemic diseases (such as diabetes mellitus, autoimmune diseases, or severe cardiovascular disease).

#### 4.2.2 Exclusion Criteria

Patients with severe hepatic or renal insufficiency, malignant tumors, or active systemic infections; patients with a history of autoimmune diseases, or those receiving long‐term immunosuppressive therapy or glucocorticoid treatment; pregnant or lactating women; patients with foot ulcers resulting from non‐diabetic causes (e.g., venous insufficiency, vasculitis, or trauma in non‐diabetic patients); patients with incomplete clinical data or unwillingness to participate in this study.

A total of 20 clinical tissue samples were collected, comprising 10 cases in the diabetic wound group and 10 cases in the acute wound/normal control group.

### 4.3 Demographic and Clinical Characteristics of the Study Cohort

**Table:** 

Group	Patient. No.	Race	Gender	Age	Wound location	Wound diameter	Wound duration
Control	1	Asian	Male	55	Chin	2.3 cm	10 h
Control	2	Asian	Female	42	Chin	4.3 cm	12 h
Control	3	Asian	Female	26	Cheek	2.5 cm	22 h
Diabetes	4	Asian	Male	35	Foot	4.6 cm	Chronic
Diabetes	5	Asian	Male	32	Foot	3.1 cm	Chronic
Diabetes	6	Asian	Female	54	Foot	5.6 cm	Chronic

### 4.4 Choline Depletion Method

To verify whether the anti‐senescence effects of Rho are specifically dependent on choline, choline depletion was performed according to a previously established enzymatic method [25]. Briefly, cells were incubated in choline‐free medium supplemented with choline oxidase and catalase. The principle of this method relies on choline oxidase to convert extracellular free choline into betaine, which cannot be re‐uptaken by cells. This enzymatic reaction establishes a concentration gradient that drives the continuous efflux and depletion of intracellular choline, thereby effectively blocking choline‐mediated signaling. Catalase was included to protect cells from H_2_O_2_ toxicity generated during the enzymatic reaction.

### 4.5 Cell Culture

C57BL/6 mice were provided by the Animal Experiment Center of Huazhong University of Science and Technology. The femur specimens were harvested, and bone marrow was flushed using a syringe with cell culture medium. The isolated bone marrow cells were cultured and induced in 1640 medium supplemented with M‐CSF cytokine at 37 °C in a 5% CO_2_ incubator. The medium was replaced on day 3, 5, and 7, and BMDM was successfully obtained by day 7.

The mice were euthanized by cervical dislocation, and the dorsal skin was harvested and immersed in 75% ethanol for 5 min. Skin samples were then placed in cold PBS containing antibiotics, followed by the removal of blood vessels and fat tissue. The samples were digested with trypsin for 1 h to isolate the dermis. After three washes with PBS, the dermal portion was minced and digested in trypsin again for 1 h at 37 °C in a water bath. The reaction was terminated by adding complete DMEM medium, and the mixture was centrifuged to discard the supernatant, leaving the tissue pellet. The pellet was then plated in a 10 cm culture dish and supplemented with 6 mL of complete medium. Cultivation was continued at 37 °C in a 5% CO_2_ incubator, with medium changes on days 3 and 5. Fibroblast was observed to migrate out of the dermis after one week, and the cells were identified and confirmed to be fibroblast.

### 4.6 Acquisition of the Optimal Drug Concentration Curve for Rho

BMDM was seeded in a 96‐well plate at a density of 1 × 10^4^ cells per well. A 100 µL aliquot of the cell suspension was added to each well of the 96‐well plate, which was then incubated in a 37 °C, 5% CO_2_ incubator for 24 h to allow cell adhesion.

Seven concentration groups (0, 5, 10, 20, 40, 80, and 160 µM) including one blank control group were set up using the “serial dilution method”. Each group was run in triplicate wells. The plate was returned to the incubator for an additional 24 h incubation.

Cell viability was determined via the CCK‐8 assay. Cell viability was calculated using the following formula: The optical density (OD) value of the blank control group (0 µmol/L) was defined as 100% viability, and the viability of other concentration groups was calculated as (OD value of experimental group / OD value of blank group) × 100%.

The mean viability ± standard deviation (Mean±SD) was calculated for each concentration group, generating a dataset with “drug concentration (x‐axis)” and “cell viability (y‐axis)”. The concentration values on the y‐axis were transformed using log_10_ (concentration).

### 4.7 BMDM Co‐Culture With Fibroblast

Co‐culture chambers with 4 µm mesh diameter were used, with macrophages in the upper layer and Fibroblast in the lower layer. The drug Rho (20 µM) and Dip (5 nM) were added to the medium of the upper chamber.

### 4.8 Immunofluorescence Staining Assay

Cells were fixed with paraformaldehyde for 20 min, followed by three washes with PBS. Subsequently, Triton was applied for 20 min, and the cells were washed three times with PBS. Goat serum was used for blocking for 30 min. The primary antibody was incubated overnight, followed by three washes with PBS. Subsequently, the fluorescently labeled secondary antibody was incubated for 30 min, followed by three washes with PBS. Finally, the cells were incubated with DAPI dye for 10 min and then mounted.

### 4.9 EdU Staining Assay

Add EdU working solution to the cells to achieve a final concentration of 10 µM and continue incubating for 2 h. Then, remove the culture medium and fix the cells with 1 mL of fixative for 15 min. Wash the cells three times with PBS. Add permeabilization solution and incubate at room temperature for 10–15 min. Remove the permeabilization solution and wash the cells 1–2 times with PBS. Subsequently, add click additive solution and incubate at room temperature in the dark for 30 min. Remove the click reaction solution and wash the cells three times with PBS, with each wash lasting 3–5 min. Stain the nuclei using Hoechst 33342; incubate at room temperature in the dark for 10 min, followed by three washes with PBS, each lasting 3–5 min. Fluorescence detection is then performed.

### 4.10 Senescence β‐Galactosidase Staining Assay

Remove the cell culture medium and wash once with PBS or HBSS. Add 1 mL of β‐galactosidase staining fixative and incubate at room temperature for 15 min. After aspirating the fixation solution, wash the cells three times with PBS. After removing PBS, add 1 mL of staining working solution to each well (preparation method is described in the instructions of Senescence β‐Galactosidase Staining Kit). Incubate overnight at 37 °C. The following day, observe under a standard optical microscope.

### 4.11 Transwell Assay

2 × 10^4^ Fibroblast suspended in FBS‐free medium were seeded into a Transwell 24‐well plate (3422, Corning Inc., USA). The lower chamber was filled with 600 µL of FBS‐containing medium as a nutritional attractant. After 24 h of incubation, the cells were washed three times with PBS, fixed with 4% formaldehyde for 30 min, and then washed three times with PBS again. Following this, the cells were stained with 0.5% crystal violet for 30 min and washed three times with PBS. Non‐invaded or non‐migrated cells were removed with a clean cotton swab. Random fields were captured from each well under a light microscope.

### 4.12 Wound Healing Assay

After digesting the cells, they were seeded into plastic molds with a width of 0.5 cm. Once the cells adhered and covered the surface of the plate, the molds were carefully removed with forceps, leaving a 0.5 cm wide wound in the cell layer. The cells were subjected to the corresponding treatments, and images were captured under a light microscope at 0 and 24 h. Data were measured and analyzed according to the scale bar.

### 4.13 Cell Cycle Assay

Cell cycle and apoptosis were detected using the Cell Cycle and Apoptosis Analysis Kit (C1052, Beyotime). Briefly, treated cells were collected and washed with pre‐cooled PBS, followed by fixation with 70% ice‐cold ethanol overnight at 4 °C. After thorough washing to remove residual ethanol, cells were incubated with RNase A at 37 °C to eliminate intracellular RNA, and then stained with propidium iodide (PI) in the dark. Subsequently, cell samples were detected by flow cytometry, and the obtained raw data were imported into FlowJo software. The cell cycle module was applied to fit and analyze the cell cycle distribution, and the proportions of cells in G0/G1, S, and G2/M phases were statistically calculated after removing cell debris and cell aggregates.

### 4.14 Western Blotting

After the cells had been washed with pre‐chilled PBS, they were added with pre‐chilled RIPA lysis buffer (Boster Biological Technology, Wuhan, China. Catalog#AR0102), Protease Inhibitor Cocktail, Phosphatase Inhibitor Cocktail I, and Phosphatase Inhibitor Cocktail II. After that, proteins were separated on a 10% SDS‐PAGE gel and then transferred to PVDF membranes. PVDF membranes were treated overnight at 4 °C with particular antibodies following a 1 h blocking step with nonfat milk. Membranes were washed three times for 10 min in TBS‐T. After being treated with secondary antibody for 1 h at room temperature, membranes were washed three times for 10 min in TBS‐T again. The membranes were treated with chemiluminescent substrate and then imaged using the Bio‐Rad Image Capture System after being washed three times with TBS‐T.

### 4.15 Quantitative Real‐Time PCR (qRT‐PCR)

Total RNA was extracted using Trizol (Invitrogen) following the manufacturer's instructions. The extraction of total RNA was assessed for purity and concentration by measuring the optical density at wavelengths of 260 and 280 nm. The purity and concentration of RNA were evaluated based on the optical density at 280 nm. Complementary DNA (cDNA) was synthesized using 1000 ng of total RNA. qRT‐PCR was performed on equal amounts of cDNA using the qPCR SYBR Green Master Mix (Yisheng Biotechnology, China), with GAPDH as the reference gene. Primers were synthesized by SeqHealth (Wuhan, China) using the following sequences.

Human:

*TP53*‐forward: CAGCACATGACGGAGGTTGT,

*TP53*‐reverse: TCATCCAAATACTCCACACGC;

*CDKN2A*‐forward:ATAGTTACGGTCGGAGGCCG,

*CDKN2A*‐reverse: TCGAAGCGCTACCTGATTCC;

*IL1B*‐forward: AACCTCTTCGAGGCACAAGG,

*IL1B*‐reverse: AGATTCGTAGCTGGATGCCG;

*IL6*‐forward: TTCGGTCCAGTTGCCTTCTCC,

*IL6*‐reverse: TTGGGTCAGGGGTGGTTATTG;

Mouse:

*Trp53*‐forward: TGCACGTATCTCCTCCCTCA,

*Trp53*‐reverse: CACCCGGATAAGATGCTGGG;

*Cdkn1a*‐forward: CCCGAGAACGGTGGAACTTT,

*Cdkn1a*‐reverse: AGAGTGCAAGACAGCGACAA;

*Cdkn2a*‐forward: GAGCGGGGACATCAAGACAT,

*Cdkn2a*‐reverse: ACTATGCTCACCTGGGCGTG;

*Il1b*‐forward: TGCCACCTTTTGACAGTGATG,

*Il1b*‐reverse: AAGGTCCACGGGAAAGACAC;

*Il6*‐forward: CGAATTCGCCCTTCCAAGAA,

*Il6*‐reverse: TCGGCAAACCTAGTGCGTTA;

### 4.16 Reactive Oxygen Species (ROS) Assay

To evaluate the intracellular total reactive oxygen species (ROS) scavenging capacity of the PLL‐*g*‐HPA hydrogel, BMDM was seeded and subjected to the indicated treatments. The probe DCFH‐DA (2',7'‐dichlorodihydrofluorescein diacetate) was employed to detect total ROS levels based on the principle that it is hydrolyzed by intracellular esterase's and subsequently oxidized by total ROS to produce green fluorescence. Briefly, BMDM was incubated with DCFH‐DA diluted in serum‐free medium at a final concentration of 10 µmol/L for 20 min at 37 °C in the dark. Following the incubation, the cells were washed three times with serum‐free medium to thoroughly remove the unincorporated extracellular probe. Fluorescence images were captured using a fluorescence microscope and the mean fluorescence intensity (MFI) was quantified using ImageJ software.

### 4.17 Cell Viability

BMDM or fibroblast was inoculated in 96‐well plates at 5 × 10^3^ cells per well. After 24 h, cells were co‐cultured with different hydrogels. Cell viability was tested by the Cytotoxicity Assay Kit. After incubation for 30 min, live or dead cells were observed under a fluorescence microscope and cell viability was also tested by Cell Counting Kit‐8. After the treatment is completed, 10 µL of CCK‐8 reagent is added to each well. The plates are then incubated for a period of time. The optical density (OD) of each well is measured at a wavelength of 450 nm using a microplate reader. The cell viability is calculated by comparing the optical density values with the control group.

### 4.18 Preparation of M‐Rho

Briefly, MSN (10 mg) was dispersed in 1 mL of methanol containing Rho (2 mg/mL) and stirred continuously at room temperature in the dark for 24 h. The Rho‐loaded MSN nanoparticles were then collected by centrifugation at 12,000 rpm for 15 min, and the supernatant was discarded. The resulting Rho@MSN precipitates were washed three times with PBS to remove unencapsulated free Rhoifolin. Membrane proteins were extracted from BMDM using a membrane protein extraction kit. Finally, the obtained MSN+Rho precipitates were resuspended in PBS and thoroughly mixed with the purified macrophage membrane vesicles. The mixture was extruded back and forth through a 400 nm polycarbonate porous membrane using an Avanti mini‐extruder (Avanti Polar Lipids, Canada) for at least 20 cycles [26]. This extrusion process facilitated the fusion of the macrophage membrane onto the surface of MSN+Rho, yielding the final macrophage membrane‐coated nanoparticles, designated as M‐Rho. The resulting M‐Rho nanoparticles were stored at 4 °C for subsequent experiments.

### 4.19 Preparation of PLL‐*g*‐HPA Hydrogel

To synthesize PLL‐*g*‐HPA, HPA (109 mg, 0.718 mmol) was first dissolved in 40 mL of DMSO, followed by activation with EDC‐HCl (414 mg, 2.16 mmol) and NHS (249 mg, 2.16 mmol) at 25 °C for 1 h. Subsequently, PLL75 (1.51 g, 7.18 mmol of NH2 groups), dissolved in 45 mL of dimethyl sulfoxide was added to the above solution and allowed to react for one day. The reaction mixture was then transferred to a dialysis tube made of seamless cellulose and purified by dialysis against deionized water. The final product, PLL‐*g*‐HPA, was obtained through freeze‐drying [27], with a theoretical grafting efficiency of 10% for HPA. Hydrogel was synthesized by Shanghai Kunhui Technology Co.

### 4.20 Physical Characterization

To obtain the microstructure of the hydrogel, we first subjected the hydrogel samples to freeze‐drying using a lyophilizer. The samples were then sealed and temporarily stored at −80 °C. Cross‐section preparation of the material was conducted, and the microstructural images of the hydrogel were acquired using SEM with a Zeiss GeminiSEM 300 (Germany).

### 4.21 Establishment of Diabetic Wound Models

For the mice model, diabetes was induced in 6‐week‐old male C57BL/6 mice (weighing 20–25 g). Mice were fed a high‐fat diet for four consecutive weeks, followed by three consecutive daily intraperitoneal injections of streptozotocin (STZ, 60 mg/kg). Mice with random blood glucose levels exceeding 16.7 mmol/L for two consecutive weeks were considered diabetic. Diabetic mice were then anesthetized with 1% pentobarbital sodium, and two uniform full‐thickness cutaneous wounds (1 cm in diameter) were created on the dorsal skin using a sterile biopsy punch.

For the beagle model, Diabetes was established in beagles (6 months old, male, weighing 10–12 kg) through a combined induction protocol of high‐fat diet feeding and pharmacological intervention. The beagles were fed a high‐fat diet for three consecutive months to induce systemic insulin resistance, followed by intravenous injections of streptozotocin (STZ, 30 mg/kg) and alloxan (ALX, 50 mg/kg) three times within one week to partially destroy pancreatic β‐cells.

Prior to induction, the baseline fasting blood glucose levels of the beagles were 4.5 ± 0.5 mmol/L. One week after the final injection, the blood glucose levels rose significantly. Beagles with fasting blood glucose levels exceeding 11.1 mmol/L for two consecutive weeks were diagnosed as diabetic and included in the subsequent experiments. During the modeling period, the body weight of the beagles remained stable (from 11.3 ± 0.7 kg to 11.7 ± 1.1 kg), indicating that the induction protocol did not cause severe cachexia or body weight loss. The successfully induced diabetic model was maintained for at least two weeks to ensure stabilization of the metabolic disorder prior to wound creation.

After confirming the stability of the diabetic model, the beagles were anesthetized with intramuscular injection of zolazepam and tiletamine. Full‐layer skin wounds (2.0 cm in length, 2.0 cm in width, and 0.5 cm in depth) were created on both sides of the dorsal region under sterile conditions. The wounds were spaced approximately 3 cm apart and protected with a sterile gauze bandage.

For the beagle experiment, the statistical unit was defined as the individual animal (*n *= 3 per group); for each beagle, the bilateral wounds received identical treatments, and the data from the two wounds were averaged to represent a single biological replicate.

### 4.22 Animals and Wound Healing and Repair Assessment in Vivo

Diabetic mice or beagle dogs were randomly divided into four groups, each undergoing the corresponding wound procedure. Photographs were taken and measurements were obtained using calipers on days 0, 3, 7, 10, and 14 post‐wound formation. The wound area percentage was calculated using ImageJ as follows:

Cn=An/×100%

where, C_n_ represents the wound area ratio, A_n_ is the wound area on day n, and A_0_ is the wound area on day 0.

### 4.23 Statistical Analysis

All data are presented as the mean ± standard deviation (SD) from at least three independent experiments. Statistical analyses were performed using GraphPad Prism software (version 9.5, GraphPad Software, USA). The normality of data distribution was assessed using the Shapiro‐Wilk test. For comparisons between two groups, statistical significance was determined using a two‐tailed, unpaired Student's t‐test. For comparisons among multiple groups, one‐way analysis of variance (ANOVA) followed by Tukey's post hoc test was applied. Statistical significance was defined as *p* < 0.05. The significance levels are indicated as follows: *p* < 0.05, *p < 0.01, and **p < 0.001.

## Author Contributions

**Jiewen Liao**: Conceptualization, methodology, software, investigation, data curation, and Writing – original draft. **Lin Gan**: Validation, formal analysis, and data curation. **Li Lu**: Conceptualization, methodology, and resources. **Lizhi Ouyang**: Investigation, data curation, and formal analysis. **Yuan Xiong**: Validation and writing – original draft. **Fawwaz Al‐Smadi**: Validationand writing – original draft. **Guandong Dai**: Validation. **Mohammad‐Ali Shahbazi**: Writing – review & editing. **Bobin Mi**: Resources, supervision, and project administration. **Mengfei Liu**: Visualization and funding acquisition. **Guohui Liu**: Conceptualization, investigation, supervision, and funding acquisition.

## Funding

The National Natural Science Foundation of China (NSFC, Grant No. 82272491). M.A.S acknowledges financial support from the University Medical Center Groningen.

## Ethics Statement

The animal study protocol was approved by Institutional Animal Care and Use Committee of the Tongji Medical College, Huazhong University of Science and Technology. IACUC Number: 3937.

The study was approved by the local Institutional Review Board (Permit number: UHCT‐IEC‐SOP‐016‐03‐01) and carried out in accordance with the Declaration of Helsinki, with written informed consent obtained from all participants.

## Conflicts of Interest

The authors declare no conflicts of interest.

## Supporting information




**Supporting File**: adhm71700‐sup‐0001‐SuppMat.docx.

## Data Availability

The data that support the findings of this study are available from the corresponding author upon reasonable request.
