# Supplementary material for: PLL‐g‐HPA Hydrogel Microneedles Loaded With Rhoifolin Target Macrophages to Alleviate Fibroblast Senescence for Diabetic Wound Healing
Source: Adv Healthc Mater. 2026 Sep 16;15(38):e71700. doi: 10.1002/adhm.71700 (PMC13674132; doi:10.1002/adhm.71700)
Supplement: Supplementary file 1 [file ADHM-15-0-s001.docx]

**PLL-g-HPA Hydrogel Microneedles Loaded with Rhoifolin Target Macrophages to Alleviate Fibroblast Senescence for Diabetic Wound Healing**

Jiewen Liao^1,2,#^, Lin Gan^1,2,#^, Li Lu^1,2,3,#^, Lizhi Ouyang^1,2,#^,Yuan Xiong^4^, Fawwaz Al-Smadi^1,2^, Guandong Dai^6^, Mohammad-Ali Shahbazi^5,^*, Bobin Mi^1,2,7^*, Mengfei Liu^1,2,^*, Guohui Liu^1,2,^*

^1^Department of Orthopedics, Union Hospital, Tongji Medical College, Huazhong University of Science and Technology, Wuhan 430022, China

^2^Hubei Province Key Laboratory of Oral and Maxillofacial Development and Regeneration, Wuhan 430022, China

^3^Department of Rehabilitation, Tongji Hospital, Tongji Medical College, Huazhong University of Science and Technology, Wuhan 430030, China

^4^Department of Orthopedics, Tongji Hospital, Tongji Medical College, Huazhong University of Science and Technology, Wuhan 430030, China

^5^Department of Biomaterials and Biomedical Technology, The Personalized Medicine Research Institute (PRECISION), University Medical Center Groningen, University of Groningen, 9713 AV Groningen, the Netherlands

^6^Department of Orthopaedics, Pingshan District People’s Hospital of Shenzhen, Pingshan General Hospital of Southern Medical University, Shenzhen, Guangdong, 518118, China

^7^Fuzhou Zhixin Future Pharmaceutical Technology Co., Ltd.,Fuzhou 350001,China

^#^These authors contributed equally to this paper

*Correspondence:

Mohammad-Ali Shahbazi, Email: [m.a.shahbazi@umcg.nl](mailto:m.a.shahbazi@umcg.nl);

Bobin Mi, E-mail: [mibobin@hust.edu.cn](mailto:mibobin@hust.edu.cn);

Mengfei Liu, E-mail: [dr_liumengfei@icloud.com](mailto:dr_liumengfei@icloud.com);

Guohui Liu, E-mail: [liuguohui@hust.edu.cn](mailto:liuguohui@hust.edu.cn)

**Figure S1. Standard curves for mouse IL-1β and IL-6 ELISA.** A) Standard curve of mouse IL-1β showing absorbance (OD450) as a function of cytokine concentration (R² > 0.99). B) Standard curve of mouse IL-6 showing absorbance (OD450) as a function of cytokine concentration (R² > 0.99).

**Figure S2. Schematic diagram of fibroblast identification and co-culture system** A) Morphological identification of fibroblast under light microscopy. B) Immunofluorescence identification of fibroblast (Vimentin and Fibronectin). C) Schematic diagram of the transwell co-culture system.

**Figure S3. Detection of the Optimal Concentration of Rho**. A) Optimal drug concentration curve. B) iNOS/Arg-1 immunofluorescence images of BMDM under treatment with different concentrations of Rho. n=3.

**Figure S4. Rhoifolin alleviates fibroblast senescence and proliferation arrest by suppressing macrophage-derived IL-1β and IL-6.** Fibroblast were co-cultured with BMDM under three conditions: IL-11, IL-11+Rhoifolin, and IL-11+Rhoifolin supplemented with exogenous IL-1β and IL-6. A) Evaluation of fibroblast proliferation by EdU incorporation assay, B) cellular senescence by SA-β-gal staining, and cell migration by C) Transwell assay and D) wound healing assay . Immunofluorescence staining of E) p21, PCNA , and F) γH2AX in fibroblast. G) Flow cytometric analysis of cell cycle distribution in fibroblast. H) Western blot analysis of the expression levels of proliferation-related proteins (PCNA, Cyclin D3, CDK2, CDK4) and senescence-related proteins (p21, p16) in fibroblast. n=3。

**Figure S5. Choline alleviates fibroblast senescence via activating the RAS/p38MAPK/p53/E2F1 pathway.** Fibroblast were co-cultured with BMDM supernatants under the indicated experimental conditions. The detailed grouping information for panels (A–I) is presented in the corresponding grouping tables . A) Western blot analysis of the RAS/p38MAPK/p53/E2F1 signaling pathway in fibroblast. Evaluation of fibroblast proliferation by B) EdU incorporation assay, cellular senescence by C) SA-β-gal staining, and cell migration by D) Transwell assay and E) wound healing assay; Immunofluorescence staining of F) p21, PCNA, and G) γH2AX; H) Flow cytometric analysis of cell cycle distribution in fibroblast. I) Western blot analysis confirming the activation status of the RAS/p38MAPK/p53/E2F1 axis in the aforementioned genetically modified fibroblast. n=3.

**Figure S6. Antioxidant and antibacterial properties of PLL-*g*-HPA hydrogel.** A) Representative fluorescence images of ROS levels in BMDM treated with IL-11 and different concentrations (5%, 10%, 15%) of PLL-*g*-HPA hydrogel and quantitative analysis of ROS fluorescence intensity. B) Schematic illustration of the antibacterial assay. C) Representative photographs of *E. coli* and *S. aureus* colonies on agar plates after incubation with PBS (Blank), 10% PLL-*g*-HPA, or 15% PLL-*g*-HPA hydrogel, and the corresponding survival rate statistics. D) Scanning electron microscopy (SEM) images of *E. coli* and *S. aureus* after different treatments, and the quantitative analysis of damaged bacteria percentages. All results are expressed as mean ± SD, n=3. ns, non-significant, * P < 0.05, **P < 0.01, ***P < 0.001.

**Figure S7. Toxicity Assessment of Materials.** A) CCK-8 assay (n=6). B) Live/dead staining of BMDM and fibroblast (n=3). C) Hemolysis assay (n=3). D) Mouse blood cell analysis (n=5). E) The biochemical analysis of liver function (n=5). F) Examination of H&E-stained sections of the heart, liver, spleen, lungs, and kidneys of diabetic mice (n=3). All results are expressed as mean ± SD, ns, non-significant, * P < 0.05, **P < 0.01, ***P < 0.001.
